# COVID19 Disease Map, a computational knowledge repository of virus–host interaction mechanisms

**DOI:** 10.15252/msb.202110387

**Published:** 2021-10-19

**Authors:** Marek Ostaszewski, Anna Niarakis, Alexander Mazein, Inna Kuperstein, Robert Phair, Aurelio Orta‐Resendiz, Vidisha Singh, Sara Sadat Aghamiri, Marcio Luis Acencio, Enrico Glaab, Andreas Ruepp, Gisela Fobo, Corinna Montrone, Barbara Brauner, Goar Frishman, Luis Cristóbal Monraz Gómez, Julia Somers, Matti Hoch, Shailendra Kumar Gupta, Julia Scheel, Hanna Borlinghaus, Tobias Czauderna, Falk Schreiber, Arnau Montagud, Miguel Ponce de Leon, Akira Funahashi, Yusuke Hiki, Noriko Hiroi, Takahiro G Yamada, Andreas Dräger, Alina Renz, Muhammad Naveez, Zsolt Bocskei, Francesco Messina, Daniela Börnigen, Liam Fergusson, Marta Conti, Marius Rameil, Vanessa Nakonecnij, Jakob Vanhoefer, Leonard Schmiester, Muying Wang, Emily E Ackerman, Jason E Shoemaker, Jeremy Zucker, Kristie Oxford, Jeremy Teuton, Ebru Kocakaya, Gökçe Yağmur Summak, Kristina Hanspers, Martina Kutmon, Susan Coort, Lars Eijssen, Friederike Ehrhart, Devasahayam Arokia Balaya Rex, Denise Slenter, Marvin Martens, Nhung Pham, Robin Haw, Bijay Jassal, Lisa Matthews, Marija Orlic‐Milacic, Andrea Senff Ribeiro, Karen Rothfels, Veronica Shamovsky, Ralf Stephan, Cristoffer Sevilla, Thawfeek Varusai, Jean‐Marie Ravel, Rupsha Fraser, Vera Ortseifen, Silvia Marchesi, Piotr Gawron, Ewa Smula, Laurent Heirendt, Venkata Satagopam, Guanming Wu, Anders Riutta, Martin Golebiewski, Stuart Owen, Carole Goble, Xiaoming Hu, Rupert W Overall, Dieter Maier, Angela Bauch, Benjamin M Gyori, John A Bachman, Carlos Vega, Valentin Grouès, Miguel Vazquez, Pablo Porras, Luana Licata, Marta Iannuccelli, Francesca Sacco, Anastasia Nesterova, Anton Yuryev, Anita de Waard, Denes Turei, Augustin Luna, Ozgun Babur, Sylvain Soliman, Alberto Valdeolivas, Marina Esteban‐Medina, Maria Peña‐Chilet, Kinza Rian, Tomáš Helikar, Bhanwar Lal Puniya, Dezso Modos, Agatha Treveil, Marton Olbei, Bertrand De Meulder, Stephane Ballereau, Aurélien Dugourd, Aurélien Naldi, Vincent Noël, Laurence Calzone, Chris Sander, Emek Demir, Tamas Korcsmaros, Tom C Freeman, Franck Augé, Jacques S Beckmann, Jan Hasenauer, Olaf Wolkenhauer, Egon L Wilighagen, Alexander R Pico, Chris T Evelo, Marc E Gillespie, Lincoln D Stein, Henning Hermjakob, Peter D'Eustachio, Julio Saez‐Rodriguez, Joaquin Dopazo, Alfonso Valencia, Hiroaki Kitano, Emmanuel Barillot, Charles Auffray, Rudi Balling, Reinhard Schneider, the COVID‐19 Disease Map Community

**Affiliations:** ^1^ Luxembourg Centre for Systems Biomedicine University of Luxembourg Esch‐sur‐Alzette Luxembourg; ^2^ Université Paris‐Saclay Laboratoire Européen de Recherche pour la Polyarthrite rhumatoïde ‐ Genhotel Univ Evry Evry France; ^3^ Lifeware Group Inria Saclay‐Ile de France Palaiseau France; ^4^ Institut Curie PSL Research University Paris France; ^5^ INSERM Paris France; ^6^ MINES ParisTech PSL Research University Paris France; ^7^ Integrative Bioinformatics, Inc. Mountain View CA USA; ^8^ Institut Pasteur Université de Paris, Unité HIV Inflammation et Persistance Paris France; ^9^ Bio Sorbonne Paris Cité Université de Paris Paris France; ^10^ Inserm‐ Institut national de la santé et de la recherche médicale Paris France; ^11^ Institute of Experimental Genetics (IEG) Helmholtz Zentrum München‐German Research Center for Environmental Health (GmbH) Neuherberg Germany; ^12^ Department of Molecular and Medical Genetics Oregon Health & Sciences University Portland OR USA; ^13^ Department of Systems Biology and Bioinformatics University of Rostock Rostock Germany; ^14^ Department of Computer and Information Science University of Konstanz Konstanz Germany; ^15^ Faculty of Information Technology Department of Human‐Centred Computing Monash University Clayton Vic. Australia; ^16^ Barcelona Supercomputing Center (BSC) Barcelona Spain; ^17^ Department of Biosciences and Informatics Keio University Yokohama Japan; ^18^ Graduate School of Media and Governance Research Institute at SFC Keio University Kanagawa Japan; ^19^ Computational Systems Biology of Infections and Antimicrobial‐Resistant Pathogens Institute for Bioinformatics and Medical Informatics (IBMI) University of Tübingen Tübingen Germany; ^20^ Department of Computer Science University of Tübingen Tübingen Germany; ^21^ German Center for Infection Research (DZIF), partner site Tübingen Germany; ^22^ Institute of Applied Computer Systems Riga Technical University Riga Latvia; ^23^ Sanofi R&D Translational Sciences Chilly‐Mazarin France; ^24^ Dipartimento di Epidemiologia Ricerca Pre‐Clinica e Diagnostica Avanzata National Institute for Infectious Diseases 'Lazzaro Spallanzani' I.R.C.C.S. Rome Italy; ^25^ COVID‐19 INMI Network Medicine for IDs Study Group National Institute for Infectious Diseases 'Lazzaro Spallanzani' I.R.C.C.S Rome Italy; ^26^ Bioinformatics Core Facility Universitätsklinikum Hamburg‐Eppendorf Hamburg Germany; ^27^ Royal (Dick) School of Veterinary Medicine The University of Edinburgh Edinburgh UK; ^28^ Faculty of Mathematics and Natural Sciences University of Bonn Bonn Germany; ^29^ Center for Mathematics Chair of Mathematical Modeling of Biological Systems Technische Universität München Garching Germany; ^30^ Department of Chemical and Petroleum Engineering University of Pittsburgh Pittsburgh PA USA; ^31^ Department of Computational and Systems Biology University of Pittsburgh Pittsburgh PA USA; ^32^ Pacific Northwest National Laboratory Richland WA USA; ^33^ Stem Cell Institute Ankara University Ankara Turkey; ^34^ Institute of Data Science and Biotechnology Gladstone Institutes San Francisco CA USA; ^35^ Department of Bioinformatics ‐ BiGCaT NUTRIM Maastricht University Maastricht The Netherlands; ^36^ Maastricht Centre for Systems Biology (MaCSBio) Maastricht University Maastricht The Netherlands; ^37^ Maastricht University Medical Centre Maastricht The Netherlands; ^38^ Center for Systems Biology and Molecular Medicine Yenepoya (Deemed to be University) Mangalore India; ^39^ MaRS Centre Ontario Institute for Cancer Research Toronto ON Canada; ^40^ NYU Grossman School of Medicine New York NY USA; ^41^ Universidade Federal do Paraná Curitiba Brasil; ^42^ European Bioinformatics Institute (EMBL‐EBI) European Molecular Biology Laboratory Hinxton, Cambridgeshire UK; ^43^ INSERM UMR_S 1256 Nutrition, Genetics, and Environmental Risk Exposure (NGERE) Faculty of Medicine of Nancy University of Lorraine Nancy France; ^44^ Laboratoire de génétique médicale CHRU Nancy Nancy France; ^45^ Queen's Medical Research Institute The University of Edinburgh Edinburgh UK; ^46^ Senior Research Group in Genome Research of Industrial Microorganisms Center for Biotechnology Bielefeld University Bielefeld Germany; ^47^ Department of Surgical Science Uppsala University Uppsala Sweden; ^48^ Institute of Computing Science Poznan University of Technology Poznan Poland; ^49^ Department of Medical Informatics and Clinical Epidemiology Oregon Health & Science University Portland OR USA; ^50^ Heidelberg Institute for Theoretical Studies (HITS) Heidelberg Germany; ^51^ Department of Computer Science The University of Manchester Manchester UK; ^52^ German Center for Neurodegenerative Diseases (DZNE) Dresden Dresden Germany; ^53^ Center for Regenerative Therapies Dresden (CRTD) Technische Universität Dresden Dresden Germany; ^54^ Institute for Biology Humboldt University of Berlin Berlin Germany; ^55^ Biomax Informatics AG Planegg Germany; ^56^ Harvard Medical School Laboratory of Systems Pharmacology Boston MA USA; ^57^ Department of Biology University of Rome Tor Vergata Rome Italy; ^58^ Elsevier Philadelphia PA USA; ^59^ Research Collaborations Unit Elsevier Jericho VT USA; ^60^ Institute for Computational Biomedicine Heidelberg University Heidelberg Germany; ^61^ cBio Center, Divisions of Biostatistics and Computational Biology Department of Data Sciences Dana‐Farber Cancer Institute Boston MA USA; ^62^ Department of Cell Biology Harvard Medical School Boston MA USA; ^63^ Computer Science Department University of Massachusetts Boston Boston MA USA; ^64^ Clinical Bioinformatics Area Fundación Progreso y Salud (FPS) Hospital Virgen del Rocio Sevilla Spain; ^65^ Computational Systems Medicine Group Institute of Biomedicine of Seville (IBIS) Hospital Virgen del Rocio Sevilla Spain; ^66^ Bioinformatics in Rare Diseases (BiER) Centro de Investigación Biomédica en Red de Enfermedades Raras (CIBERER) FPS, Hospital Virgen del Rocío Sevilla Spain; ^67^ Department of Biochemistry University of Nebraska‐Lincoln Lincoln NE USA; ^68^ Quadram Institute Bioscience Norwich UK; ^69^ Earlham Institute Norwich UK; ^70^ European Institute for Systems Biology and Medicine (EISBM) Vourles France; ^71^ Cancer Research UK Cambridge Institute University of Cambridge Cambridge UK; ^72^ Institute of Experimental Medicine and Systems Biology Faculty of Medicine, RWTH Aachen University Aachen Germany; ^73^ The Roslin Institute University of Edinburgh Edinburgh UK; ^74^ University of Lausanne Lausanne Switzerland; ^75^ Helmholtz Zentrum München – German Research Center for Environmental Health Institute of Computational Biology Neuherberg Germany; ^76^ Interdisciplinary Research Unit Mathematics and Life Sciences University of Bonn Bonn Germany; ^77^ St. John’s University College of Pharmacy and Health Sciences Queens NY USA; ^78^ Department of Molecular Genetics University of Toronto Toronto ON Canada; ^79^ FPS/ELIXIR‐es Hospital Virgen del Rocío Sevilla Spain; ^80^ Institució Catalana de Recerca i Estudis Avançats (ICREA) Barcelona Spain; ^81^ Systems Biology Institute Tokyo Japan; ^82^ Okinawa Institute of Science and Technology Graduate School Okinawa Japan

**Keywords:** computable knowledge repository, large‐scale biocuration, omics data analysis, open access community effort, systems biomedicine, Computational Biology, Microbiology, Virology & Host Pathogen Interaction

## Abstract

We need to effectively combine the knowledge from surging literature with complex datasets to propose mechanistic models of SARS‐CoV‐2 infection, improving data interpretation and predicting key targets of intervention. Here, we describe a large‐scale community effort to build an open access, interoperable and computable repository of COVID‐19 molecular mechanisms. The COVID‐19 Disease Map (C19DMap) is a graphical, interactive representation of disease‐relevant molecular mechanisms linking many knowledge sources. Notably, it is a computational resource for graph‐based analyses and disease modelling. To this end, we established a framework of tools, platforms and guidelines necessary for a multifaceted community of biocurators, domain experts, bioinformaticians and computational biologists. The diagrams of the C19DMap, curated from the literature, are integrated with relevant interaction and text mining databases. We demonstrate the application of network analysis and modelling approaches by concrete examples to highlight new testable hypotheses. This framework helps to find signatures of SARS‐CoV‐2 predisposition, treatment response or prioritisation of drug candidates. Such an approach may help deal with new waves of COVID‐19 or similar pandemics in the long‐term perspective.

## Graphical abstract

COVID‐19 Disease Map is a large‐scale collection of curated computational models and diagrams of molecular mechanisms involved in SARS‐CoV‐2 infection. The map supports the computational exploration of pathways affected by the virus.

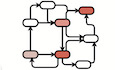

## Introduction

The coronavirus disease 2019 (COVID‐19) pandemic due to severe acute respiratory syndrome coronavirus 2 (SARS‐CoV‐2) has already resulted in the infection of over 250 million people worldwide, of whom almost 5 million have died (https://covid19.who.int, accessed on 05.10.2021). This global challenge motivated researchers worldwide to an unprecedented effort towards understanding the pathology to treat and prevent it. To date, over 170 thousand articles have been published in relation to COVID‐19 (PubMed query “covid‐19[Title/Abstract] or sars‐cov‐2[Title/Abstract]”, accessed on 01.07.2021). The reported molecular pathophysiology that links SARS‐CoV‐2 infection to the clinical manifestations and course of COVID‐19 is complex and spans multiple biological pathways, cell types and organs (Gagliardi *et al*, 2020). Resources such as Protein Data Bank repository of viral protein structures (preprint: Lubin *et al*, 2020) or the IMEx coronavirus interactome (Perfetto *et al*, 2020) offer detailed information about particular viral proteins and their direct binding partners. However, the scope of this information is limited. To gain insight into the large network of molecular mechanisms, knowledge from the vast body of scientific literature and bioinformatic databases needs to be integrated using systems biology standards. A repository of such computable knowledge will support data analysis and predictive modelling.

With this goal in mind, we initiated a collaborative effort involving over 230 biocurators, domain experts, modellers and data analysts from 120 institutions in 30 countries to develop the COVID‐19 Disease Map (C19DMap), an open access collection of curated computational diagrams and models of molecular mechanisms implicated in the disease (Ostaszewski *et al*, 2020). The C19DMap is a constantly evolving resource, refined and updated by ongoing biocuration, sharing and analysis efforts. Currently, it is a collection of 42 diagrams containing 1,836 interactions between 5,499 elements, supported by 617 publications and preprints. The summary of diagrams available in the C19DMap can be found online (https://covid.pages.uni.lu/map_contents) and in Table EV1.

In the article, we explain the effort of our multidisciplinary community to construct the interoperable content of the resource, involving biocurators, domain experts and data analysts. We introduce the scope of the C19DMap and the insight it brings into the crosstalk and regulation of COVID‐19‐related molecular mechanisms. Next, we outline analytical workflows that can be used on the contents of the map, including the initial outcomes of two case studies. We conclude with a discussion on the utility and perspectives of the C19DMap as a disease‐relevant computational repository.

## Results

### An interoperable repository of comprehensive and computable diagrams

We constructed a comprehensive diagrammatic description of disease mechanisms in a way that is both human‐ and machine‐readable, lowering communication barriers between experimental and computational biologists. To this end, we aligned the biocuration efforts of the Disease Maps Community (Mazein *et al*, 2018), Reactome (Jassal *et al*, 2020), and WikiPathways (Slenter *et al*, 2018) and developed guidelines for building and annotating these diagrams. In addition, we integrated relevant knowledge from public repositories (Licata *et al*, 2020; Perfetto *et al*, 2020; Rodchenkov *et al*, 2020; Türei *et al*, 2021) and text mining resources to update and refine the contents of the C19DMap based on other knowledge‐building efforts. This work resulted in a series of pathway diagrams constructed *de novo*, describing key events in the COVID‐19 infectious cycle and host response.

**Figure 1:**
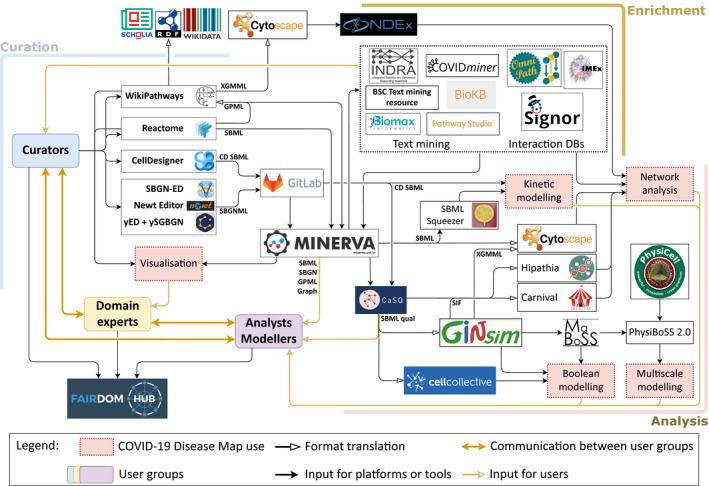
Ecosystem of the COVID‐19 Disease Map Community The main groups of the C19DMap Community are biocurators, domain experts, and analysts and modellers; communicating to refine, interpret, and apply C19DMap diagrams. These diagrams are created and maintained by biocurators, following pathway database workflows or stand‐alone diagram editors, and reviewed by domain experts. The content is shared via pathway databases or a GitLab repository; all can be enriched by integrated resources of text mining and interaction databases. The C19DMap diagrams are available in several layout‐aware systems biology formats and integrated with external repositories, allowing a range of computational analyses, including network analysis and Boolean, kinetic or multiscale simulations.

The C19DMap project involved three main groups of participants: the biocurators, the domain experts, and the analysts and modellers. Biocurators developed a collection of systems biology diagrams focused on the molecular mechanisms of SARS‐CoV‐2. Domain experts refined the contents of the diagrams using interactive visualisation and annotations. Analysts and modellers developed computational workflows to generate hypotheses and predictions about the mechanisms encoded in the diagrams. Figure 1 illustrates the ecosystem of the C19DMap Community, highlighting the roles of the participants, available format conversions, interoperable tools and downstream uses. The community members and their contributions are listed on FAIRDOMHub (Wolstencroft *et al*, 2017).

#### Creating and accessing the diagrams

The biocurators of the C19DMap diagrams followed the guidelines developed by the Community, WikiPathways (Slenter *et al*, 2018) and Reactome (Jassal *et al*, 2020) based on systems biology standards (Le Novère *et al*, 2009; Demir *et al*, 2010; Keating *et al*, 2020) and persistent identifiers (Wimalaratne *et al*, 2018). The diagrams are composed of biochemical reactions and interactions (altogether called interactions) between different molecular entities in various cellular compartments. As multiple teams worked on related topics, biocurators reviewed other diagrams, also across platforms (see also Materials and Methods). The diagrams are accessible online and can be explored using an intuitive user interface. Table 1 summarises information about the curated diagrams, and Table EV1 lists the diagrams and provides links to access them.

**Table 1:** COVID‐19 Disease Map contents.

	Source		
	Individual diagrams	Reactome	WikiPathways
Diagram contents	21 diagrams 1,334 interactions 4,272 molecular entities 397 publications	2 diagrams 101 interactions 489 molecular entities 227 publications	19 diagrams 401 interactions 738 molecular entities 61 publications
Access	GitLab gitlab.lcsb.uni.lu/covid/models	SARS‐CoV‐1 and SARS‐CoV‐2 infections collection reactome.org/PathwayBrowser/#/R‐HSA‐9679506	COVID pathway collection covid.wikipathways.org
Exploration	The MINERVA Platform (Gawron *et al*, 2016) covid19map.elixir‐luxembourg.org Guide: covid.pages.uni.lu/minerva‐guide	Native web interface Guide: covid.pages.uni.lu/reactome‐guide	Native web interface Guide: covid.pages.uni.lu/wikipathways‐guide
Biocuration guidelines	Community^a^	Platform‐specific^b^	Platform‐specific^c^
Diagram Editors	CellDesigner (Matsuoka *et al*, 2014), Newt^d^, SBGN‐ED (Czauderna *et al*, 2010), yEd+ySBGN^e^	Reactome pathway editor^b^	PathVisio (Kutmon *et al*, 2015)
Formats	CellDesigner SBML SBGNML (Bergmann *et al*, 2020)	Internal, SBML and SBGNML compliant	GPML (Kutmon *et al*, 2015)

The table summarises biocuration resources and content of the C19DMap across three main parts of the repository. All diagrams are listed in Table EV1, and available online at: https://covid.pages.uni.lu/map_contents.

^a^ https://fairdomhub.org/documents/661

^b^ https://reactome.org/community/training

^c^ https://www.wikipathways.org/index.php/Help:Editing_Pathways

^d^ https://newteditor.org

^e^ https://github.com/sbgn/ySBGN

#### Enrichment using knowledge from databases and text mining

The knowledge of COVID‐19 mechanisms is rapidly evolving, as shown by the growth of the COVID‐19 Open Research Dataset (CORD‐19), a source of manuscripts and metadata on COVID‐19‐related research (preprint: Lu Wang *et al*, 2020). CORD‐19 currently contains almost 480,000 articles and preprints, over ten times more than when it was introduced more than a year ago (accessed on 05.10.2021). In such a quickly evolving environment, manual biocuration needs to be supported by automated procedures to identify and prioritise crucial articles, molecules and their interactions to be included in the C19DMap.

Potential knowledge sources for such assisted biocuration are interaction and pathway databases, especially those with dedicated COVID‐19 content (Licata *et al*, 2020; Perfetto *et al*, 2020). Their structured and annotated information on protein interactions or causal relationships was generated using separate biocuration guidelines and formats. Nevertheless, their comparable identifiers and references to source publications make them plausible building blocks for constructing the C19DMap (see Materials and Methods).

Text mining approaches are another source of information that can direct the biocurators towards the most recent and relevant findings. They automatically extract and annotate biomolecule names and their interactions from abstracts, full‐text documents or pathway figures (Bauch *et al*, 2020; Hanspers *et al*, 2020). Networks of molecule interactions constructed by text mining can carry substantially more noise than the contents of interaction databases but offer broader literature coverage.

Table 2 summarises open access interaction databases and text mining knowledge bases supporting the biocuration of the C19DMap. Molecular interactions from these sources have a broad coverage at the cost of depth of mechanistic representation. The biocurators used this content to build and update the map by manual exploration or by programmatic comparison. First, the biocurators visually explored the contents of such networks using available search interfaces to identify interactions of interesting molecules and encoded them in the diagrams. This task was supported by a dedicated visualisation tool COVIDminer (https://rupertoverall.net/covidminer). The biocurators also used assistant chatbots that respond to natural language queries and return meaningful answers extracted from text mining platforms. Second, we developed routines for programmatic queries of the interaction databases, providing automated and reproducible exploration of the selected databases. This was realised using data endpoints: Application programming interfaces (API) for INDRA, AILANI or Pathway Studio, and SPARQL for BioKB. This automated exploration retrieved functions, interactions, pathways or drugs associated with submitted queries, e.g. gene lists. This way, otherwise time‐consuming tasks such as an assessment of completeness of a given diagram or search for new literature evidence were automated. Section “Exploration of the networked knowledge” describes an application of such automated queries in crosstalk analysis.

**Table 2:** Resources supporting biocuration of the COVID‐19 Disease Map.

Resource	Type	Manually curated	Directed	Layout	COVID‐19 specific
IMEx Consortium (Orchard *et al*, 2012)	Interaction database	Yes	No	No	Yes^a^
SIGNOR 2.0 (Licata *et al*, 2020)		Yes	Yes	Yes	Yes^b^
OmniPath (Türei *et al*, 2016)		No	Yes	No	No
Elsevier Pathway Collection^c^	Pathway	Yes	Yes	Yes	Yes^d^
INDRA (Gyori *et al*, 2017)	Text mining	Yes	Yes	No	Yes^e^
BioKB^f^		No	Yes	No	Yes
AILANI COVID‐19^g^		No	Yes	No	Yes
OpenNLP+GNormPlus^h^		No	Yes	No	Yes

They include (i) collections of COVID‐19 interactions and pathways, (ii) interaction databases and (iii) text mining corpora.

^a^ https://www.ebi.ac.uk/intact/resources/datasets#coronavirus

^b^ https://signor.uniroma2.it/covid/

^c^ https://pathwaystudio.com

^d^ http://dx.doi.org/10.17632/d55xn2c8mw.1

^e^ https://emmaa.indra.bio/dashboard/covid19

^f^ https://biokb.lcsb.uni.lu

^g^ https://ailani.ai

^h^ https://gitlab.lcsb.uni.lu/covid/models/‐/tree/master/Resources/Text%20mining

#### Interoperability of the diagrams and annotations

The biocuration of the C19DMap diagrams was distributed across multiple teams, using varying tools and associated systems biology representations. This required a common approach to annotations of diagram elements and their interactions. Additionally, to compare and combine the diagrams in the C19DMap, interoperability of layout‐aware formats was needed.

The diagrams were encoded in three layout‐aware formats for standardised representation of molecular interactions: SBML, SBGNML and GPML. All three formats, centred around molecular interactions, provided a constrained vocabulary to encode element and interaction types, encode layout of corresponding diagrams and support stable identifiers for diagram components. These shared properties, supported by a common ontology (Courtot *et al*, 2011), allowed cross‐format translation of the diagrams, which was essential for harmonising the effort between biocuration platforms.

The ecosystem of tools and resources supporting the C19DMap (see Fig 1) ensured interoperability between SBML, SBGNML and GPML via translation, preserving the diagram layout (Bohler *et al*, 2016; Balaur *et al*, 2020; Hoksza *et al*, 2020) for harmonised visualisation of diagrams. Additionally, these diagrams were transformed into inputs of computational pipelines and data repositories, allowing network analysis, pathway modelling and interoperability with molecular interaction repositories (Pillich *et al*, 2017) (see Materials and Methods).

### Structure and scope of the COVID‐19 Disease Map

**Figure 2:**
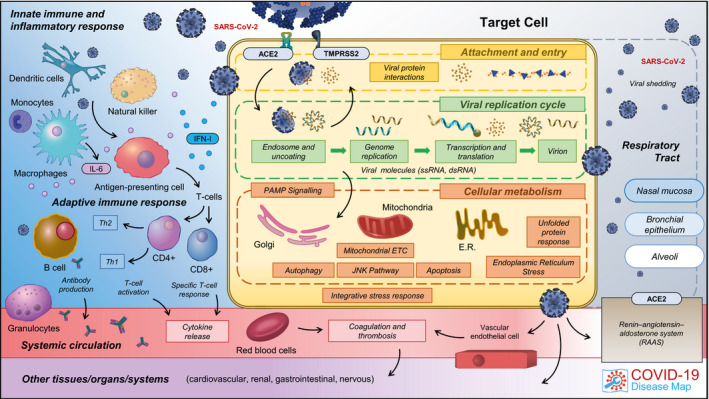
The structure and content of the COVID‐19 Disease Map An overview of the areas of focus of the C19DMap biocuration. Go to covid19map.elixir‐luxembourg.org for an interactive version. Full list of diagrams and browsing instructions are available online at covid.pages.uni.lu/map_contents.

The C19DMap was built bottom‐up, exploiting a rich bioinformatics framework discussed in Section “An interoperable repository of comprehensive and computable diagrams” of the Results, based on knowledge from existing studies of other coronaviruses (Fung & Liu, 2019) and contextualised with data emerging from studies of SARS‐CoV‐2 (Gordon *et al*, 2020). The contents of the C19DMap are available online, summarised in a constantly updated overview at https://covid.pages.uni.lu/map_contents (see also Table EV1). Currently, the C19DMap focuses on molecular processes involved in SARS‐CoV‐2 entry and replication and host–virus interactions (see Fig 2). Emerging scientific evidence of host susceptibility, immune response, cell and organ specificity will be incorporated into the next versions in accordance with our curation roadmap (https://fairdomhub.org/documents/907).

While the interactions of SARS‐CoV‐2 with various host cell types are vital determinants of COVID‐19 pathology (Hui *et al*, 2020; Mason, 2020; Ziegler *et al*, 2020), the current C19DMap represents an infection of a generic host cell. Several pathways included in the map are shared between different cell types; for example, the IFN‐1 pathway is active in dendritic and lung epithelial cells and in alveolar macrophages (Hadjadj *et al*, 2020; Lee & Shin, 2020; Sa Ribero *et al*, 2020). Continued annotations of emerging expression datasets (Delorey *et al*, 2021) and other sources of information will allow the construction of cell‐specific versions of the C19DMap to provide an integrated view of the effects of SARS‐CoV‐2 on the human organism. An example workflow to construct such a focused version of the map was proposed in Section “Case study: analysis of cell‐specific mechanisms using single‐cell expression data”.

**Figure 3:**
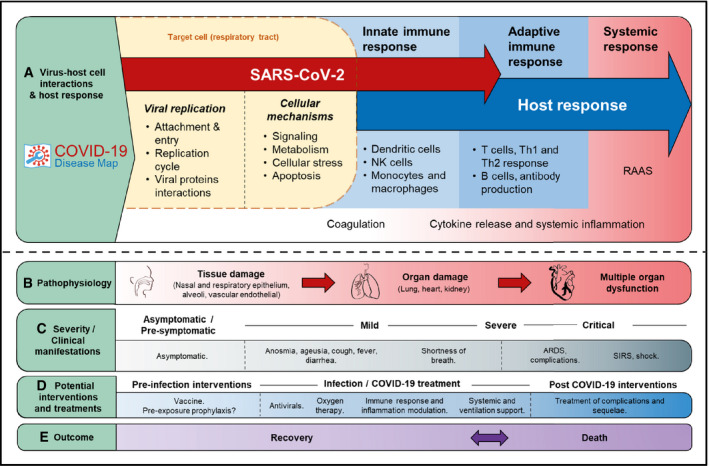
Overview of the C19DMap in the context of COVID‐19 progression The figure summarises the main sections and content of the C19DMap by illustrating the progressive but overlapping mechanisms at different levels and study features of the disease intended as quick references for the map.
Cellular level (light yellow), the immune response (blue) and other systemic responses (red) of the host following SARS‐CoV‐2 infection.The progression of pathophysiology from tissue damage to organ damage and multiple organ dysfunction in severe cases.Clinical manifestations, depending on the severity of the infection from asymptomatic to critical COVID‐19.Potential intervention strategies that may be suggested based on the analysis of the C19DMap before, during and after infection, depending on the type and target of the intervention.Clinical outcomes of SARS‐CoV‐2 infection. ARDS, acute respiratory distress syndrome. RAAS, renin–angiotensin–aldosterone system. SIRS, systemic inflammatory response syndrome. For the literature on clinical manifestations, see Lauer *et al*, 2020; He *et al*, 2020; Huang *et al*, 2020; Bajema *et al*, 2020; preprint: Chen *et al*, 2020b; Wang *et al*, 2020a; Tong *et al*, 2020.

SARS‐CoV‐2 infection and COVID‐19 progression are sequential events that start with viral attachment and entry (Fig 3). These events involve various dynamic processes and different timescales that are not captured in static representations of pathways. The correlation of symptoms and potential drugs suggested to date helps downstream data exploration and drug target interpretation in the context of therapeutic interventions.

#### Contents of the COVID‐19 Disease Map

##### Virus replication cycle

The virus replication cycle includes binding of the spike surface glycoprotein (S) to angiotensin‐converting enzyme 2 (ACE2) facilitated by TMPRSS2 (Hoffmann *et al*, 2020b; Letko *et al*, 2020) and other receptors (preprint: Amraei *et al*, 2020; preprint: Gao *et al*, 2020). Viral entry occurs either by direct fusion of the virion with the cell membranes or by endocytosis (Hoffmann *et al*, 2020a; Xia *et al*, 2020) of the virion membrane and the subsequent injection of the nucleocapsid into the cytoplasm. Within the host cell, the C19DMap depicts how SARS‐CoV‐2 hijacks the rough endoplasmic reticulum (RER)‐linked host translational machinery for its replication (Chen *et al*, 2010; Angelini *et al*, 2013; Nakagawa *et al*, 2016; V’kovski *et al*, 2019). The RER‐attached translation machinery produces structural proteins, which, together with the newly generated viral RNA, are assembled into new virions and released to the extracellular space via smooth‐walled vesicles (Nakagawa *et al*, 2016) or hijacked lysosomes (Ghosh *et al*, 2020).

These mechanisms are illustrated in the diagrams of the “Virus replication cycle” section in Table EV1: “Attachment and entry”, “Transcription, translation and replication” and “Assembly and release”.

##### Viral subversion of host defence

Endoplasmic reticulum (ER) stress results from the production of large amounts of viral proteins that create an overload of unfolded proteins (Krähling *et al*, 2009; DeDiego *et al*, 2011; Fukushi *et al*, 2012). The mechanisms of the unfolded protein response (UPR) include the mitigation of the misfolded protein load by reduced protein synthesis and increased protein degradation (Sureda *et al*, 2020) through the ubiquitin–proteasome system (UPS) and autophagy (Choi *et al*, 2018; Bello‐Perez *et al*, 2020). SARS‐CoV‐2 may perturb the process of UPS‐based protein degradation via the interaction of the viral Orf10 protein with the Cul2 ubiquitin ligase complex and its putative substrates (Gordon *et al*, 2020; Zhang *et al*, 2020). The involvement of SARS‐CoV‐2 in autophagy is less documented (Yang & Shen, 2020).

The increased burden of misfolded proteins due to viral replication and subversion of mitigation mechanisms may trigger programmed cell death (apoptosis). The C19DMap encodes major signalling pathways triggering this final form of cellular defence against viral replication (Diemer *et al*, 2010). Many viruses block or delay cell death by expressing anti‐apoptotic proteins to maximise the production of viral progeny (Kanzawa *et al*, 2006; Liu *et al*, 2007) or induce it in selected cell types (Diemer *et al*, 2010; Chu *et al*, 2016; preprint: Chen *et al*, 2020b).

These mechanisms are illustrated in the diagrams of the “Viral subversion of host defence” section in Table EV1: “ER stress and unfolded protein response”, “Autophagy and protein degradation” and “Apoptosis”.

##### Host integrative stress response

Severe acute respiratory syndrome coronavirus 2 infection damages the epithelium and the pulmonary capillary vascular endothelium (Bao *et al*, 2020), impairing respiration and leading to acute respiratory distress syndrome (ARDS) in severe forms of COVID‐19 (Huang *et al*, 2020). The release of pro‐inflammatory cytokines and hyper‐inflammation are known complications, causing further widespread damage (Chen *et al*, 2020a; Lucas *et al*, 2020). Coagulation disturbances and thrombosis are associated with severe cases, but specific mechanisms have not been described yet (Iba *et al*, 2020; Klok *et al*, 2020). Nevertheless, it was shown that SARS‐CoV‐2 disrupts the coagulation cascade and causes renin–angiotensin system (RAS) imbalance (Magro *et al*, 2020; Urwyler *et al*, 2020).

Angiotensin‐converting enzyme 2, used by SARS‐CoV‐2 for host cell entry, is a regulator of RAS and is widely expressed in the affected organs. The diagrams in the repository describe how ACE2‐converted angiotensins trigger the counter‐regulatory arms of RAS and the downstream signalling via AGTR1, regulating the coagulation cascade (Gheblawi *et al*, 2020; McFadyen *et al*, 2020).

These mechanisms are illustrated in the diagrams of the “Integrative stress response” section in Table EV1: “Renin–angiotensin system” and “Coagulopathy”.

##### Host immune response

The innate immune system detects specific pathogen‐associated molecular patterns, through pattern recognition receptors (PRRs) that recognise viral RNA in the endosome during endocytosis or in the cytoplasm during virus replication. The PRRs activate associated transcription factors promoting the production of antiviral proteins such as interferon‐alpha, interferon‐beta and interferon‐lambda (Takeuchi & Akira, 2010; Berthelot & Lioté, 2020; Blanco‐Melo *et al*, 2020; Hadjadj *et al*, 2020; Park & Iwasaki, 2020). SARS‐CoV‐2 impairs this mechanism (Chu *et al*, 2020), but the exact components are yet to be elucidated (Liao *et al*, 2005; Devaraj *et al*, 2007; Frieman *et al*, 2007; Li *et al*, 2016; Bastard *et al*, 2020). The C19DMap includes both the virus recognition process and the viral evasion mechanisms. It provides the connection between virus entry, its replication cycle, and the effector pathways of pro‐inflammatory cytokines, especially of the interferon type I cascade (Wong *et al*, 2018; Mesev *et al*, 2019; Mantlo *et al*, 2020; Su & Jiang, 2020; Thoms *et al*, 2020; Ziegler *et al*, 2020).

Key metabolic pathways modulate the availability of nutrients and critical metabolites of the immune microenvironment (Rao *et al*, 2019). They are a target of infectious agents that reprogram host metabolism to create favourable conditions for their reproduction (Kedia‐Mehta & Finlay, 2019). The C19DMap encodes several immunometabolic pathways and provides detailed information about the way SARS‐CoV‐2 proteins interact with them. The metabolic pathways include haem catabolism (Batra *et al*, 2020) and its downstream target, the NLRP3 inflammasome (van den Berg & Te Velde, 2020), tryptophan‐kynurenine metabolism governing the response to inflammatory cytokines (Murakami *et al*, 2013; preprint: Su *et al*, 2020), and nicotinamide and purine metabolism (Renz *et al*, 2020). Finally, we represent the pyrimidine synthesis pathway, tightly linked to purine metabolism, affecting viral DNA and RNA syntheses (Hayek *et al*, 2020; Xiong *et al*, 2020).

These mechanisms are illustrated in the diagrams of the “Innate Immune Response” section in Table EV1: “PAMP signalling”, “Induction of interferons and the cytokine storm” and “Altered host metabolism”.

#### Exploration of the networked knowledge

**Figure 4:**
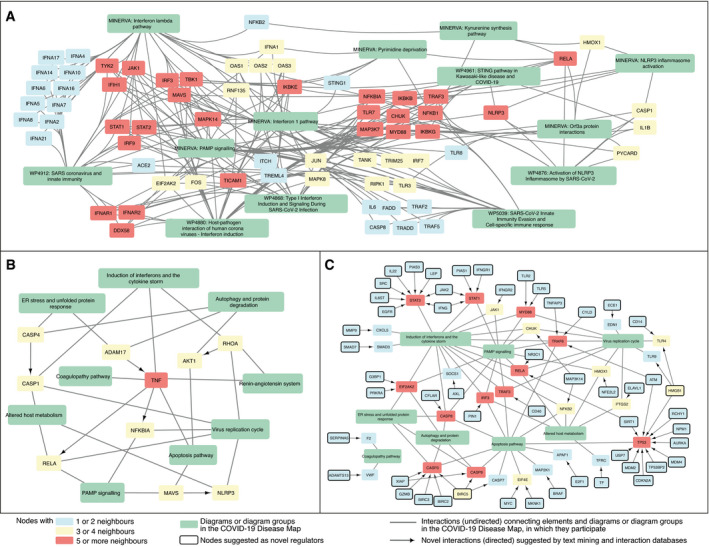
Exploration of the existing and candidate crosstalk between the diagrams of the COVID‐19 Disease Map The network structure of the diagrams and their interactions based on existing crosstalk (shared elements), candidate crosstalk, and candidate regulators. Colour code: green—pathways or pathway groups; blue—proteins with one or two neighbours; yellow—proteins with three or four neighbours; and red—proteins with five or more neighbours. Candidate molecular interactions are shown as directed edges. Candidate regulator elements are marked with a solid black border. See Materials and Methods for details.
Existing crosstalk between individual diagrams of IFN‐I and RELA‐related mechanisms.Candidate crosstalk between pathway groups.Candidate regulators of existing diagrams from text mining and interaction databases.

The diagrams of the C19DMap were curated in a distributed manner across various platforms and tools. In order to coordinate such an effort and get a systematic overview of the contents of the map, we programmatically analysed the content of the diagrams, benefiting from their standard encoding and annotation (see Materials and Methods). This allowed us to identify crosstalk and functional overlaps across pathways. Then, we linked the diagrams to interaction and text mining databases to fill the gaps in our understanding of COVID‐19 mechanisms and generate new testable hypotheses. Below, we discuss three specific examples of exploration of this networked knowledge (see Fig 4). Access to the complete content of the crosstalk diagrams can be found in Materials and Methods.

##### Existing crosstalk between COVID‐19 Disease Map diagrams

**Figure EV1:**
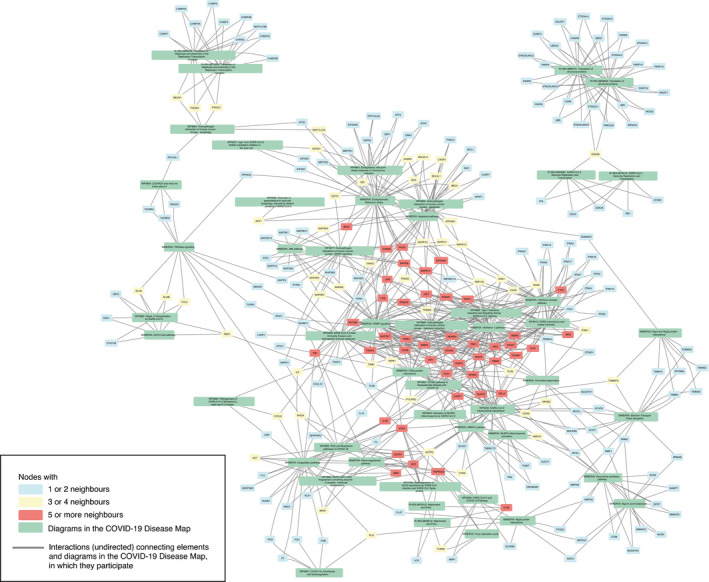
Exploration of the existing crosstalk between the diagrams of the COVID‐19 Disease Map The network structure of the diagrams and their interactions based on existing crosstalk (shared elements). Colour code: green—pathways; blue—proteins with one or two neighbours; yellow—proteins with three or four neighbours; and red—proteins with five or more neighbours. See Materials and Methods for details.

**Figure EV2:**
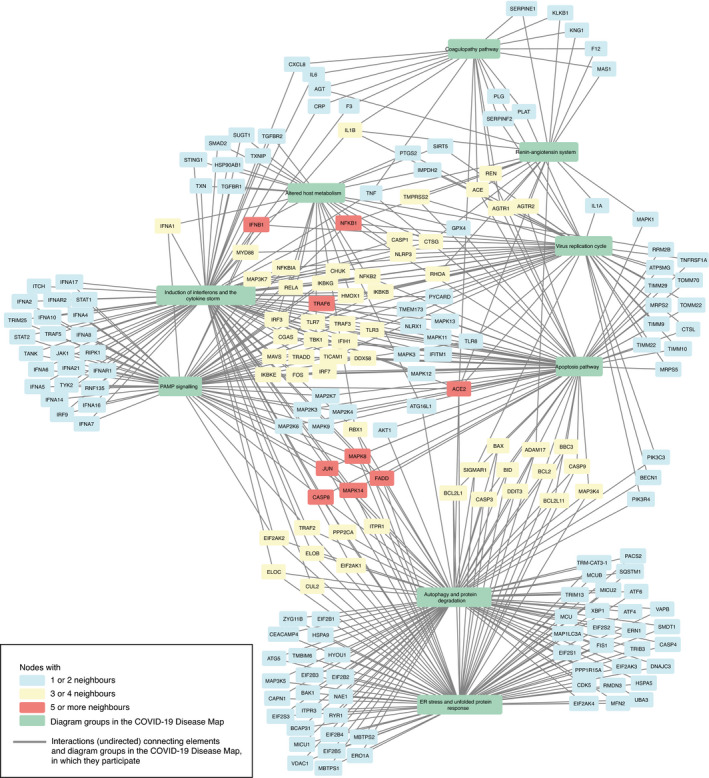
Exploration of the existing crosstalk between the groups of diagrams of the COVID‐19 Disease Map The network structure of the diagrams and their interactions based on existing crosstalk (shared elements). Colour code: green—pathway groups; blue—proteins with one or two neighbours, yellow—proteins with three or four neighbours; and red—proteins with five or more neighbours. See Materials and Methods for details.

First, the existing pathways crosstalk emerged by matching entities between the diagrams (Figs 4A, EV1 and EV2). For instance, they link different pathways involved in type I IFN (IFN‐1) signalling. Responses to RNA viruses and pathogen‐associated molecular patterns (PAMPs) share common pathways, involving RIG‐I/Mda‐5, TBK1/IKKE and TLR signalling, leading to the production of IFN‐1s, especially IFN‐beta (Häcker & Karin, 2006) and IFN‐alpha (Mogensen, 2009). Downstream, IFN‐1 activates Tyk2 and Jak1 protein tyrosine kinases, causing STAT1:STAT2:IRF9 (ISGF3) complex formation to promote the transcription of IFN‐stimulated genes (ISGs). Importantly, TBK1 also phosphorylates IKBA, an NF‐kB inhibitor, for proteasomal degradation in crosstalk with the UPS pathway, allowing free NF‐kB and IRF3 to co‐activate ISGs (Fang *et al*, 2017). Another TBK1 activator, STING, links IFN signalling with pyrimidine metabolism.

SARS‐CoV‐2 M protein affects these IFN responses by inhibiting the RIG‐I:MAVS:TRAF3 complex and TBK1, preventing IRF3 phosphorylation, nuclear translocation and activation (Zheng *et al*, 2020). In severe COVID‐19 cases, elevated NF‐kB activation associated with impaired IFN‐1 (Hadjadj *et al*, 2020) may be a host attempt to compensate for the lack of IFN‐1 activation (Rubio *et al*, 2013), leading to NF‐kB hyperactivation and release of pro‐inflammatory cytokines. Also, SARS‐CoV‐1 viral papain‐like proteases, contained within the nsp3 and nsp16 proteins, inhibit STING and its downstream IFN secretion (Chen *et al*, 2014). Perturbations in these pathways may impair the IFN response against SARS‐CoV‐2 and explain persistent blood viral load and an exacerbated inflammatory response in COVID‐19 patients (Hadjadj *et al*, 2020).

##### New crosstalk from interaction and text mining datasets

**Figure EV3:**
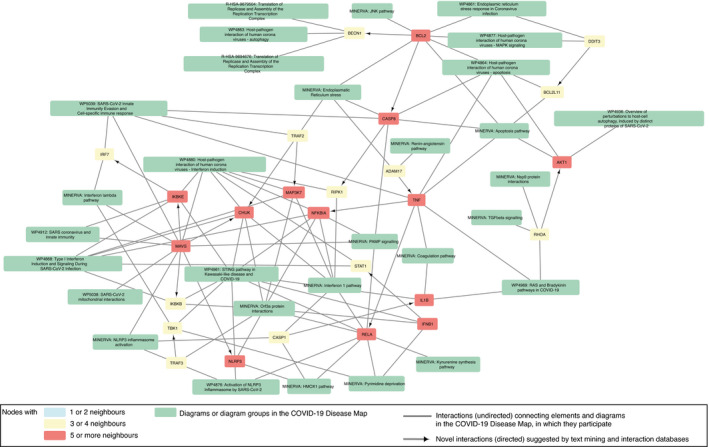
Exploration of new crosstalk between the diagrams of the COVID‐19 Disease Map The network structure of the diagrams and their interactions based on new crosstalk. Colour code: green—pathways; blue—proteins with one or two neighbours; yellow—proteins with three or four neighbours; and red—proteins with five or more neighbours. New molecular interactions are shown as directed edges. See Materials and Methods for details.

New relationships emerging from associated interaction and text mining databases (see Section “Exploration of the networked knowledge” of the Results) suggested new pathway crosstalk (see Figs 4B and EV3). One of these was the interplay between ER stress and the immune pathways, as PPP1R15A regulates the expression of TNF and the translational inhibition of both IFN‐1 and IL‐6 (Smith, 2018). This finding coincided with the proposed interaction of pathways responsible for protein degradation and viral detection, as SQSTM1, an autophagy receptor and NFKB1 regulator, controls the activity of cGAS, a double‐stranded DNA detector (Seo *et al*, 2018). Another association revealed by text mining data was ADAM17 and TNF release from the immune cells in response to ACE2‐S protein interaction with SARS‐CoV‐1 (Haga *et al*, 2008), potentially increasing the risk of COVID‐19 infection (Zipeto *et al*, 2020). This new interaction connected diagrams of the (i) “Viral replication cycle” via ACE2‐S protein interactions, (ii) “Viral subversion of host defence mechanisms” via ER stress, (iii) “Host integrative stress response” via the renin–angiotensin system and (iv) “Host innate immune response” via pathways implicating TNF signalling.

##### Novel regulators of protein activity

Finally, we identified potential novel regulators of proteins in the C19DMap using interaction and text mining databases (see Fig 4C). These proteins take no part in the current version of the map but interact with molecules already represented in at least one of the diagrams. An example of such a novel regulator was NFE2L2, which controls the activity of HMOX1 in the context of viral infection (Kesic *et al*, 2011). In turn, HMOX1 controls immunomodulatory haem metabolism (Zhang *et al*, 2019), the mechanisms of viral replication, and is a target of SARS‐CoV‐2 Orf3a protein (Miao *et al*, 2020). The suggested NFE2L2‐HMOX1 interaction is supported by the literature reports of NFE2L2 importance in COVID‐19 cardiovascular complications due to crosstalk with the renin–angiotensin signalling pathway (Valencia *et al*, 2020) and potential interactions with viral entry mechanisms (Hassan *et al*, 2020). Interestingly, the modulation of the NFE2L2‐HMOX1 axis was already proposed as a therapeutic measure for inflammatory diseases (Attucks *et al*, 2014), making it an appealing extension of the C19DMap.

### Computational analysis and modelling for hypothesis generation

The standardised representation and programmatic access to the contents of the C19DMap support reproducible analytical and modelling workflows. Here, we discuss the range of possible approaches and demonstrate preliminary results, focusing on interoperability, reproducibility and applicability of the methods and tools. Table 3 summarises selected computational workflows that can support data interpretation and hypothesis testing in COVID‐19 research.

**Table 3:** Examples of computational workflows using the COVID‐19 Disease Map

Workflow	COVID‐19 Disease Map contents	User input	Tools	Output
Data interpretation	Online diagrams	Transcriptomics, Proteomics, Metabolomics	The MINERVA Platform (Gawron *et al*, 2016) PathVisio (Kutmon *et al*, 2015) Reactome (Jassal *et al*, 2020)	Visualisation of SARS‐CoV‐2 mechanisms contextualised to data^a^
	Diagrams in SIF format (via CasQ)	Transcriptomics	DoRothEA (Garcia‐Alonso *et al*, 2019)	Contextualised evaluation of transcription factors activity under SARS‐CoV‐2 infection^b^
	Diagrams in SIF format (via CasQ)	Interactome data Proteomics	Network clustering (Messina *et al*, 2020)	Identification of new SARS‐CoV‐2‐relevant interactions
Mechanistic modelling	Diagrams in SIF format (via CasQ)	TranscriptomicsMetabolomics	HiPATHIA (Salavert *et al*, 2016) CARNIVAL (Liu *et al*, 2019)	Endpoint prediction^c^ Drug target effect prediction
Discrete modelling	Diagrams in SBML qual format (via CasQ)	Perturbation hypothesis (loss/gain of function)	CellCollective (Helikar *et al*, 2012) GINSim (Naldi *et al*, 2018a) BoolNet	Perturbation outcomes:^c^ ‐ Real‐time simulation ‐ Attractor analysis
Stochastic & Multiscale modelling	Diagrams in SBML qual format (via CasQ)	Perturbation hypothesis (loss/gain of function)	PhysiBoSS (Letort *et al*, 2019): MaBoSS (Stoll *et al*, 2017) + PhysiCell (Ghaffarizadeh *et al*, 2018)	Perturbation outcomes:^d^ ‐ Real‐time simulation ‐ Stochastic multiscale modelling

Each workflow relies on the input from the C19DMap, either a direct diagram or its transformed contents, available in the GitLab repository. The workflow users may supply omics datasets to interpret them in the context of the map or test their hypotheses about how disease models will behave under specific perturbations.

^a^ Example: see Results, Case study—analysis of cell‐specific mechanisms using single‐cell expression data.

^b^ Example: see Results, Case study—RNA‐Seq‐based analysis of transcription factor activity.

^c^ Example: see Results, Case study—RNA‐Seq‐based analysis of pathway signalling.

^d^ https://colomoto.github.io/colomoto‐docker/

#### Data interpretation and network analysis

The projection of omics data onto the C19DMap broadens and deepens our understanding of disease‐specific mechanisms, in contrast to classical pathway enrichment analyses, which often produce lists of generic biological mechanisms. Visualisation of omics datasets on the map diagrams creates overlays, allowing interpretation of specific conditions, such as disease severity or cell types (Satagopam *et al*, 2016).

Datasets projected on the C19DMap can create signatures of molecular regulation determined by the expression levels of the corresponding molecules. Together, multiple omics readouts and multiple measurements can increase the robustness of such signatures (De Meulder *et al*, 2018). This interpretation can be extended using available SARS‐CoV‐2‐related omics and interaction datasets (Bouhaddou *et al*, 2020) to infer which transcription factors, their target genes and signalling pathways are affected upon infection (Dugourd & Saez‐Rodriguez, 2019). Combining regulatory interactions of the C19DMap with such data collections extends the scope of the analysis and may suggest new mechanisms to include in the map.

Besides the visual exploration of omics datasets, the network structure of the C19DMap allows extended network analysis of viral–human protein–protein interactions (PPIs) (Gordon *et al*, 2020). It can be expanded by merging virus–host with human PPIs and proteomics data to discover clusters of interactions indicating human biological processes affected by the virus (Messina *et al*, 2020). These clusters can be interpreted by visualising them on the C19DMap diagrams to reveal additional pathways or interactions to add to the map.

#### Mechanistic and dynamic computational modelling

Diagrams from the C19DMap can be coupled with omics datasets to estimate their functional profiles and predict the effect of interventions, e.g. effects of drugs on their targets (Salavert *et al*, 2016). However, such an approach has a substantial computational complexity, limiting the size of the input diagrams. Large‐scale mechanistic pathway modelling can address this challenge but requires transformation of diagrams into causal networks, which, combined with transcriptomics, (phospho‐)proteomics or metabolomics data (Dugourd *et al*, 2021), contextualise the networks and hypotheses about intervention outcomes. Both approaches provide a set of coherent causal links connecting upstream drivers such as stimulations or pathogenic mutations to downstream changes in diagram endpoints or transcription factor activities.

Dynamic modelling allows analysis of changes of molecular networks in time to understand their complexity under disease‐related perturbations (Naldi *et al*, 2018b). C19DMap diagrams, translated to SBML qual using CaSQ (see Materials and Methods), can be used in discrete modelling, using modelling software that supports SBML qual file import. Notably, multiscale processes involved in viral infection, from molecular interactions to multicellular behaviour, can be simulated using a dedicated computational architecture. In such a multiscale setup, single‐cell models run in parallel to capture the behaviour of heterogeneous cell populations and their intercellular communications at different time scales, e.g. diffusion, cell mechanics, cell cycle, or signal transduction (Osborne *et al*, 2017; preprint: Wang *et al*, 2020). Implementing detailed COVID‐19 signalling models in the PhysiBoSS framework (Letort *et al*, 2019) may help better understand complex dynamics of interactions between immune system components and the host cell.

#### Case study: analysis of cell‐specific mechanisms using single‐cell expression data

To investigate cell‐specific mechanisms of COVID‐19, we projected single‐cell expression data onto the C19DMap. To this end, we calculated differentially expressed genes (DEGs) for two datasets relevant to the disease. The first dataset describes non‐infected bronchial secretory cells (Lukassen *et al*, 2020; Data ref: Lukassen *et al*, 2020), where we selected DEGs from three different subtypes of secretory cells dubbed (i) secretory1, (ii) secretory2 and (iii) secretory3 (transient) cells. The second dataset describes SARS‐CoV‐2‐infected intestinal organoids (Triana *et al*, 2021; Data ref: Triana *et al*, 2021), where we selected DEGs from (iv) infected and (v) bystander immature enterocytes from the intestinal organoids infected with SARS‐CoV‐2. DEGs (i), (ii) and (iii) can serve as an illustration of pathway activity across normal lung cells, while datasets (iv) and (v) demonstrate a comparison of molecular activity in cells of infected intestinal tissue. These selected datasets are available as overlays in the C19DMap and can be interactively explored, showing cell‐type‐specific dysregulation of particular diagrams (see Materials and Methods).

Visual exploration of the differential expression profiles in the C19DMap revealed that transient secretory cells specifically express molecules associated with the virus replication cycle (TMPRSS2). This suggests that these cells are more susceptible to viral entry than the other types of bronchial secretory cells. Also, the interferon 1 signalling pathway was up‐regulated in both secretory1 and transient secretory cells. However, transient secretory cells showed up‐regulation of elements up‐ and downstream of the pathway (IFNAR1‐JAK1, and ISG15 or OAS1); in secretory1 cells, the up‐regulated proteins were downstream (transcription factor AP‐1). In the intestinal organoid dataset, the comparison of infected and bystander immature enterocytes confirmed the downregulation of the ACE2 receptor reported by the original article (Triana *et al*, 2021; Data ref: Triana *et al*, 2021), as visualised in the virus replication cycle diagram. In addition, exploration of other affected pathways may suggest the context of this observation – for instance, the C19DMap demonstrated the differential activity of the pyrimidine deprivation pathway, which could suggest a reduction of transcriptional activity as a host response to the viral infection. Enrichment analysis of diagrams indicated that mitochondrial dysfunction, apoptosis, and inflammasome activation were dysregulated in infected enterocytes. The enrichment analysis of the cell‐type‐specific overlays was obtained by the GSEA plugin of the C19DMap. These results can be replicated and examined directly by the users via the visual interface of the C19DMap (see https://covid.pages.uni.lu/minerva‐guide/ and Materials and Methods).

#### Case study: RNA‐Seq‐based analysis of transcription factor activity

As discussed above, the diagrams of the C19DMap can be coupled with omics datasets. Here, we highlight how the map systematically reveals the transcription factors (TFs) related to SARS‐CoV‐2 infection. To do so, we conducted differential expression analysis between SARS‐CoV‐2 infected Calu‐3 human lung adenocarcinoma cell line and controls. Results were used to estimate TF activity deregulation upon viral infection. We mapped the outcomes of the TF activities to pathway diagrams of the C19DMap (see Materials and Methods).

**Figure 5:**
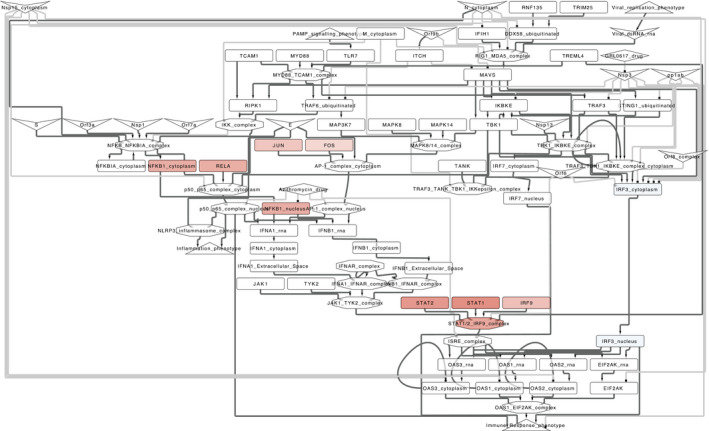
*Interferon type I signalling pathway* diagram of the COVID‐19 Disease Map integrated with TF activity derived from transcriptomics data after SARS‐CoV‐2 infection A zoom was applied in the area containing the most active TFs (red nodes) after infection. Node shapes: host genes (rectangles), host molecular complex (octagons), viral proteins (V shape), drugs (diamonds) and phenotypes (triangles).

The results for the interferon type I signalling diagram are shown in Fig 5. This pathway included some of the most active TFs after SARS‐CoV‐2 infection, such as STAT1, STAT2, IRF9 and NFKB1. These are well‐known components of cytokine signalling and antiviral responses (Cheon *et al*, 2013; Fink & Grandvaux, 2013). Interestingly, these TFs were located downstream of various viral proteins (*E*, *S*, *Nsp1*, *Orf7a* and *Orf3a*) and members of the MAPK pathway (*MAPK8*, *MAPK14* and *MAP3K7*). SARS‐CoV‐2 infection is known to promote MAPK activation, which mediates the cellular response to pathogenic infection and promotes the production of pro‐inflammatory cytokines (Bouhaddou *et al*, 2020). Overall, these results highlighted that the molecular mechanisms of the response of the human cells to SARS‐CoV‐2 infection can be investigated by combining omics datasets with the diagrams of the C19DMap.

#### Case study: RNA‐Seq‐based analysis of pathway signalling

The diagrams of the C19DMap allow for a complex analysis of how the infection may affect signalling sequences in encoded pathways based on available omics data. To demonstrate this approach, we applied a mechanistic modelling algorithm that estimates the functional profiles of signalling circuits in the context of omics datasets. We used expression profiles from nasopharyngeal swabs of COVID‐19 patients and controls (Lieberman *et al*, 2020; Data ref: Lieberman *et al*, 2020) to calculate the differential expression profiles and derive the pathway signalling activities (see Materials and Methods).

**Figure 6:**
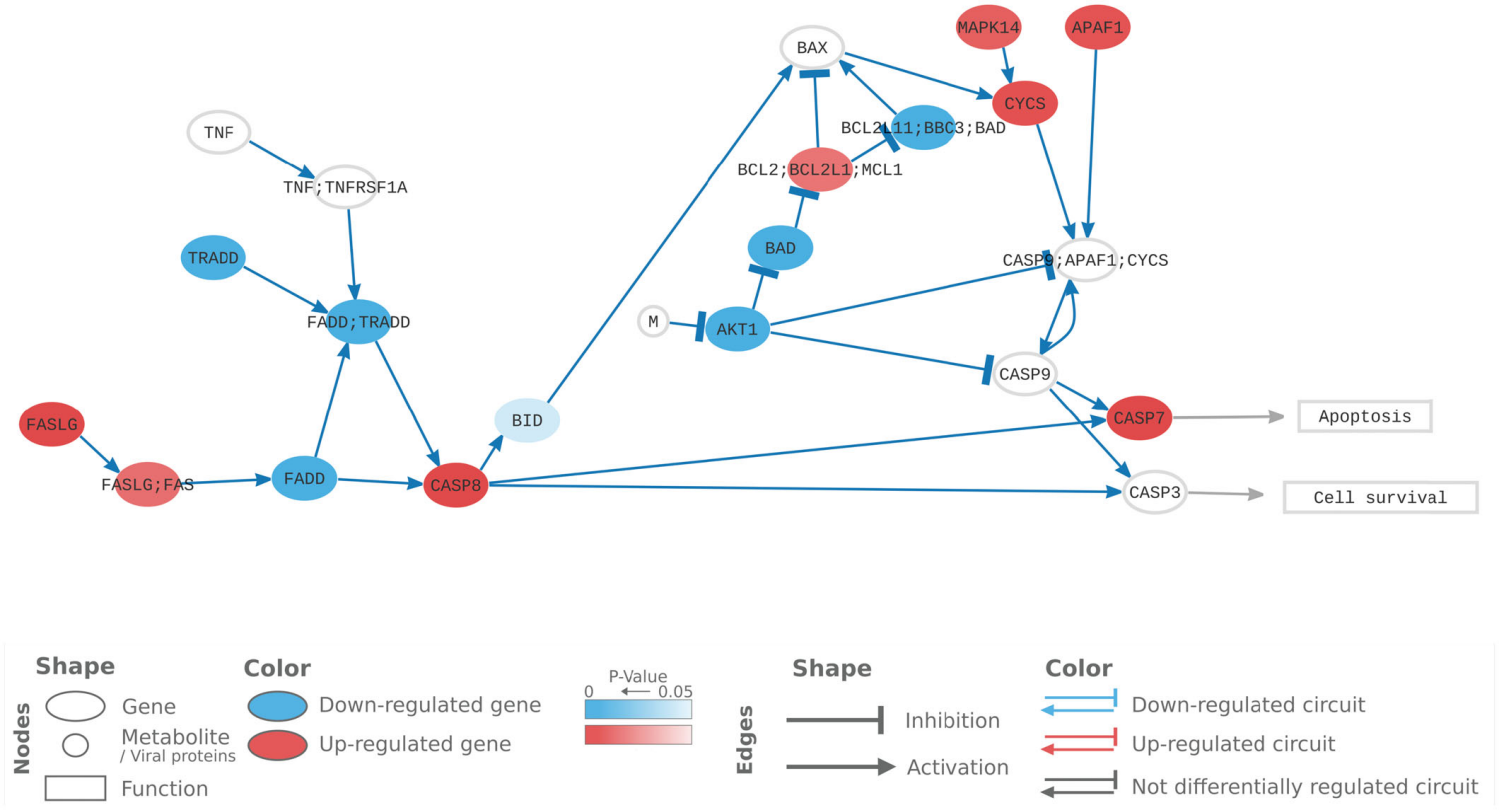
Representation of the activation levels of apoptosis pathway in nasopharyngeal swabs from SARS‐CoV‐2‐infected individuals Activation levels were calculated using transcriptional data from GSE152075 and the Hipathia mechanistic pathway analysis algorithm. Each node represents a gene (ellipse), a human metabolite/viral protein (circle) or a function (rectangle). The pathway is composed of circuits from a receptor to an effector. Significant differential regulation of circuits in infected cells is highlighted by colour arrows (blue: inactive in infected cells). The colour of elements corresponds to the level of differential expression in SARS‐CoV‐2‐infected human nasopharyngeal swabs versus non‐infected nasopharyngeal human swabs. Blue: downregulated, red: up‐regulated and white: no statistically significant differential expression.

To illustrate this approach, we focused on the results of the analysis of the apoptosis pathway, also shown in Fig 6 and Table EV2. We observed an overall downregulation of both the CASP3 and CASP7 subpathways and an inhibition of the circuit ending in effector protein CASP3, possibly due to the downregulation of AKT1 and BAD and the downstream inhibition of BAX. Although the BAX downstream genes were up‐regulated, the signal arriving at them was diminished by the effect of the previous nodes. Although CASP8 was up‐regulated, the cumulative effect of the individual node activities resulted in the inhibition of CASP7. Indeed, inflammatory response via CASP8 has been described as a result of SARS‐CoV‐2 infection (Li *et al*, 2020), and the role of caspase‐induced apoptosis has been established, together with the ripoptosome/caspase‐8 complex, as a pro‐inflammatory checkpoint (Chauhan *et al*, 2018), which may be triggering up‐regulation of such processes in other pathways. Overall, our findings recapitulate reported outcomes and provide explanations of the effects of interactions on pathway elements.

## Discussion

Our knowledge of COVID‐19 molecular mechanisms is growing at a great speed, fuelled by global research efforts to investigate the pathophysiology of SARS‐CoV‐2 infection. Keeping an overview of all the findings, many of which focus on individual molecules, is a great challenge just one year after the start of the pandemic. The C19DMap aggregates this knowledge into molecular interaction diagrams, making it available for visual exploration by life science and clinical researchers and analysis by computational biologists.

The map complements and interfaces with other COVID‐19 resources such as interaction databases (Licata *et al*, 2020; Perfetto *et al*, 2020), protein‐centric resources (preprint: Lubin *et al*, 2020) and relevant omics data repositories (Delorey *et al*, 2021) by providing a context to particular pieces of information and helping with data interpretation. The diagrams of the C19DMap describe molecular mechanisms of COVID‐19, grounded in the relevant published SARS‐CoV‐2 research, completed where necessary by mechanisms discovered in related beta‐coronaviruses.

We developed the contents of the C19DMap *de novo* in an unprecedented, community‐driven effort involving independent biocurators, as well as WikiPathway and Reactome biocurators. Over forty diagrams with molecular resolution have been constructed since March 2020, shared across three platforms. In this work, we combined and harmonised expertise in biocuration across multiple teams, formulated clear guidelines and cross‐reviewed the outcomes of our work with domain experts. Although the approach of community curation was applied in the past (Slayden *et al*, 2013; Naithani *et al*, 2019), we are not aware of any curation effort on a similar scale for a single human disease to date.

In this work, we established a computational framework accompanying the biocuration process, integrating interaction databases and text mining solutions to accelerate diagram building. This allowed us not only to enrich particular diagrams but also to explore crosstalk between them and prioritise key novel regulators of the encoded pathways. Thanks to the interoperability of different systems biology formats, we performed this analysis for diagrams constructed in different biocuration environments, extending current advances in pathway interoperability (Bohler *et al*, 2016).

Moreover, by developing reproducible analysis pipelines for the contents of the C19DMap, we promoted early harmonisation of formats, support of standards and transparency in all steps. Preliminary results of such efforts are illustrated in the case studies above. Notably, the biocurators and domain experts participated in the analysis helped to evaluate the outcomes and corrected the curated content if necessary. This way, we improve the quality of the analysis and increase the reliability of the models used to generate testable predictions.

The C19DMap is an open access repository of diagrams and reproducible workflows for content conversion and analysis. We followed FAIR principles in making our content and code available to the entire research community (Wilkinson *et al*, 2016). Importantly, FAIRDOMHub is an essential platform for disseminating all information about the project and linking contributors to their contributions. The C19DMap Community is open and expanding as more people with complementary expertise join forces. Using the FAIR approach for sharing the results of our work makes this effort more scalable. Recognising individual contributions and open access policy promote the distributed knowledge building and generation of research data.

The project aims to provide the tools to deepen our understanding of the mechanisms driving the infection and help boost drug development supported by testable suggestions. It offers insights into the dynamic nature of the disease at the molecular level and its propagation at the systemic level. Thus, it provides a platform for a precise formulation of models, accurate data interpretation, the potential for disease mitigation and drug repurposing. In the longer run, the constantly growing C19DMap content will be used to facilitate the finding of robust signatures related to SARS‐CoV‐2 infection predisposition, disease evolution or response to various treatments, along with the prioritisation of new potential drug targets or drug candidates.

This approach to an emerging worldwide pandemic leveraged the capacity and expertise of an entire swath of the bioinformatics community, bringing them together to improve the way we build and share knowledge. By aligning our efforts, we strive to provide COVID‐19‐specific pathway models, synchronise content with similar resources and encourage discussion and feedback at every stage of the curation process. Such an approach may help to deal with new waves of COVID‐19 or similar pandemics in the long‐term perspective.

## Materials and Methods

### Reagents and Tools table

**Table:** 

Reagent/Resource	Reference or Source	Identifier or Catalog Number
**Software**		
CellDesigner v4.4.2	http://www.celldesigner.org (Matsuoka *et al*, 2014)	
Newt v3.0	https://newteditor.org	
SBGN‐ED	Czauderna *et al* (2010)	
ySBGN	https://github.com/sbgn/ySBGN	
The MINERVA Platform v15.1.4	https://minerva‐web.lcsb.uni.lu (Gawron *et al*, 2016)	
Reactome	https://reactome.org (Jassal *et al*, 2020)	
WikiPathways	https://www.wikipathways.org (Slenter *et al*, 2018)	
PathVisio v3.3.0	https://pathvisio.github.io (Kutmon *et al*, 2015)	
INDRA	Gyori *et al* (2017)	
AILANI COVID‐19	https://ailani.ai	
BioKB	https://biokb.lcsb.uni.lu/topic/DOID:0080599	
OpenNLP + GNormPlus	https://opennlp.apache.org/ (Wei *et al*, 2015)	
COVIDminer	https://rupertoverall.net/covidminer	
rWikipathways v 1.12	10.18129/B9.bioc.rWikiPathways	
OmniPathR	https://github.com/saezlab/OmnipathR	
The MINERVA Conversion API v15.1	https://minerva.pages.uni.lu/doc/api/15.1/converter/ (Hoksza *et al*, 2020)	
cd2sbgml	https://github.com/sbgn/cd2sbgnml (Balaur *et al*, 2020)	
rnef2sbgn	https://github.com/golovatenkop/rnef2sbgn	
Seurat v4.0	https://satijalab.org/seurat/ (Hao *et al*, 2021)	
DESeq2	10.18129/B9.bioc.DESeq2 (Love *et al*, 2014)	
Viper v1.26.0	10.18129/B9.bioc.viper (Alvarez *et al*, 2016)	
DoRothEA v1.4.1	10.18129/B9.bioc.dorothea (Garcia‐Alonso *et al*, 2019)	
CaSQ v0.9.11	Aghamiri *et al* (2020)	
CoV‐HiPathia	Rian *et al* (2021)	
**Datasets**		
IMEx Consortium COVID‐19 dataset	Perfetto *et al* (2020)	
SIGNOR 2.0 COVID‐19 dataset	Licata *et al* (2020)	
OmniPath	Türei *et al* (2021)	
INDRA EMMAA Collection, accessed: 2020.12.01	https://emmaa.indra.bio/dashboard/covid19	
RNA‐Seq transcriptomic single‐cell profiles	https://eils‐lung.cells.ucsc.edu https://doi.org/10.6084/m9.figshare.11981034.v1 (Lukassen *et al*, 2020)	
SARS‐CoV‐2‐infected intestinal organoids	Triana *et al* (2021)	GSE156760
SARS‐CoV‐2‐infected Calu‐3 cells	Blanco‐Melo *et al* (2020)	GSE147507
SARS‐CoV‐2 nasopharyngeal swabs	Lieberman *et al* (2020)	GSE152075

### Methods and Protocols

#### Biocuration platforms

Individual diagrams were encoded in systems biology layout‐aware formats (see below) by biocurators using CellDesigner (Matsuoka *et al*, 2014), Newt (https://newteditor.org), SBGN‐ED (Czauderna *et al*, 2010) and ySBGN (https://github.com/sbgn/ySBGN). This community‐based curation was coordinated by sharing curation topics, e.g. relevant pathways or particular SARS‐CoV‐2 proteins across the community to cover the available literature and identify synergies. Curation guidelines (https://fairdomhub.org/documents/661) were established to ensure proper representation and annotation of the key features of the diagrams. Curation guidelines for logical models (Niarakis *et al*, 2020) were followed. Regular technical reviews of the diagrams were performed following a previously established checklist to harmonise their content. The diagrams are stored and versioned in a GitLab repository (https://gitlab.lcsb.uni.lu/covid/models). Individual diagrams are visualised in the MINERVA Platform (Gawron *et al*, 2016). The entry‐level view is based on Fig 2.

Reactome (Jassal *et al*, 2020) biocuration efforts initially focused on SARS‐CoV‐1 and its proteins, and their functions are extensively documented in the experimental literature. Reactome curators were assigned a subpathway from the viral life cycle, a host pathway or potential therapeutics. Curators were supported by an editorial manager and a dedicated SARS literature triage process. The resulting set of pathways for SARS‐CoV‐1 provided the basis for computational inference of the corresponding SARS‐CoV‐2 pathways based on structural and functional homologies between the two viruses. The computationally inferred SARS‐CoV‐2 infection pathway events and entities were then reviewed and manually curated using published SARS‐CoV‐2 experimental data. Reactome diagrams are available via a dedicated pathway collection (https://reactome.org/PathwayBrowser/#/R‐HSA‐9679506).

The WikiPathways (Slenter *et al*, 2018) diagrams were constructed using PathVisio (Kutmon *et al*, 2015), with annotation of pathway elements from the integrated BridgeDb identifier mapping framework (van Iersel *et al*, 2010). All pathways are stored in GPML format (Kutmon *et al*, 2015). The WikiPathways diagrams are available via a dedicated pathway portal, grouping pathway models specific to SARS‐CoV‐2, other coronaviruses and general cellular processes relevant to the virus–host interactions (https://www.wikipathways.org/index.php/Portal:COVID‐19).

#### Layout‐aware systems biology formats

The diagrams are available in SBML format (Keating *et al*, 2020), allowing computational modelling of biological processes. SBML stores visual information about encoded elements and reactions using render (Bergmann *et al*, 2018) and layout (Gauges *et al*, 2015) packages. An early version of SBML adapted by CellDesigner allows storing layout and rendering information. Systems Biology Graphical Notation (SBGN) format is a graphical standard for visual encodings of molecular entities and their interactions, implemented using SBGNML (Bergmann *et al*, 2020) for encoding the layout of SBGN maps and their annotations. Finally, GPML (Kutmon *et al*, 2015) is a structured XML format for computable representation of biological knowledge used by the WikiPathways platform.

Interactions and interacting entities are annotated following a uniform, persistent identification scheme, using either MIRIAM Registry or Identifiers.org (Juty *et al*, 2012) and the guidelines for annotations of computational models. Viral protein interactions are explicitly annotated with their taxonomy identifiers to highlight findings from strains other than SARS‐CoV‐2. Stable protein complexes from SARS‐CoV‐2 and SARS are annotated using the Complex Portal.

#### Interaction databases

The biocuration process was supported by interaction and pathway databases storing structured, annotated and curated information about COVID‐19 virus–host interactions. The IMEx Consortium (Meldal *et al*, 2019) dataset (Perfetto *et al*, 2020) contains curated Coronaviridae‐related interaction data from reviewed manuscripts and preprints, resulting in a dataset of roughly 7,300 interactions extracted from over 250 publications, including data from SARS‐CoV‐2, SARS, CoV, and other strains of Coronaviridae. The dataset is updated with every release of IMEx data and is open access (https://www.ebi.ac.uk/intact/resources/datasets#coronavirus). The SIGNOR 2.0 (Licata *et al*, 2020) dataset contains manually annotated and validated signalling interactions related to the host–virus interaction, including cellular pathways modulated during SARS‐CoV‐2 infection. The dataset was constructed from the literature on causal interactions between SARS‐CoV‐2, SARS‐COV‐1, MERS proteins and the human host and is openly available (https://signor.uniroma2.it/covid/). The Elsevier Pathway Collection (Daraselia *et al*, 2004; Nesterova *et al*, 2020) COVID‐19 dataset comprises manually reconstructed and annotated pathway diagrams. Statements about molecular interactions are extracted into a knowledge graph by a dedicated text mining technology adapted for extracting facts about viral proteins and viruses from the literature. These interactions were filtered for experimental evidence, used for pathway reconstruction and made openly available (http://dx.doi.org/10.17632/d55xn2c8mw.1). Information from OmniPath (Türei *et al*, 2021) on existing interactions gathered from pathway and interaction databases was used in a programmatic way to suggest cell‐specific interactions and cell–cell interactions specific to immune reactions.

#### Text and figure mining

Text mining was performed on the CORD‐19: COVID‐19 Open Research Dataset dataset (preprint: Lu Wang *et al*, 2020). INDRA (Gyori *et al*, 2017), AILANI COVID‐19 (https://ailani.ai) and BioKB processed CORD‐19 dataset (https://biokb.lcsb.uni.lu/topic/DOID:0080599), with their results available programmatically via REST API and SPARQL interfaces. An OpenNLP‐based (https://opennlp.apache.org/) text mining workflow using GNormPlus (Wei *et al*, 2015) was applied to the CORD‐19 dataset and the collection of MEDLINE abstracts associated with the genes in the SARS‐CoV‐2 PPI network (Gordon *et al*, 2020) using the Entrez GeneRIFs, https://www.ncbi.nlm.nih.gov/gene/about‐generif. (https://gitlab.lcsb.uni.lu/covid/models/‐/tree/master/Resources/Text%20mining). Also, we used data from 221 CORD‐19 dataset figures using a dedicated Figure Mining Workflow (Hanspers *et al*, 2020), with results available at https://gladstone‐bioinformatics.shinyapps.io/shiny‐covidpathways. Results of text mining were accessed by the curators in the form of molecular interactions with references to the articles and to sentences from which these interactions were derived. We systematically aligned the C19DMap with assembled INDRA Statements, both to enrich and to extend the map (see “Crosstalk analysis” below). The content of INDRA and AILANI COVID‐19 was accessible via interfaces that allow users to provide natural language queries, such as “What are COVID‐19 risk factors?” or “What are the interactors of ACE2?”, facilitating extracting knowledge from the results of text mining workflows. The results of the INDRA workflow were visualised using the COVIDminer project (https://rupertoverall.net/covidminer). Each extracted statement describes a directed interaction between two gene products, small molecules or biological processes. The causal network representing the COVIDminer database is browsable through a web interface. The results of the OpenNLP‐based text mining workflow were imported into a BioKC biocuration platform for structured processing and SBML export.

#### Crosstalk analysis

Crosstalk analysis was performed for the list of C19DMap diagrams (Table EV1). The code is available at: https://gitlab.lcsb.uni.lu/covid/models/‐/tree/master/Resources/Crosstalks. Individual diagrams were accessed via the API of the MINERVA Platform, WikiPathway diagrams via the rWikipathways package (https://github.com/wikipathways/rWikiPathways) and Reactome diagrams via the Reactome API. Text mining interactions are from the INDRA EMMAA Collection (https://emmaa.indra.bio/dashboard/covid19), dataset timestamp: 2020‐12‐01‐21‐05‐54. Verified molecular interactions for quality control of the text mining data were obtained from OmniPath using the OmnipathR package (https://github.com/saezlab/OmnipathR). We filtered text mining interactions of the EMMAA dataset for “belief” of 0.8 or higher and retained those matching the direction and interacting molecules to the OmniPath dataset. We call this filtered group of interactions “EMMAA‐OP interactions”.

Crosstalk between C19DMap diagrams was calculated based on the HGNC identifiers of their elements. For simplification, all elements of the same diagram were considered to be interacting with each other. Three types of networks were constructed: existing crosstalk, new crosstalk and new regulators. Diagram groups followed the scheme in the list of C19DMap diagrams (Table EV1). The networks were visualised using Cytoscape (Shannon *et al*, 2003). The colour code is common for the networks: light green for nodes representing a diagram or a diagram group, light blue for nodes having one or two neighbours, yellow for nodes having three or four neighbours and red for nodes with five or more neighbours. Diagram nodes have prefixes indicating their provenance. Diagram groups have no prefixes, as they combine diagrams across platforms. Existing crosstalk between diagrams, or groups of diagrams, was calculated by identifying shared HGNC identifiers linking diagrams or groups of diagrams. To calculate new crosstalk between diagrams, we merged the EMMAA‐OP interactions with the network of existing crosstalk and kept only those new interactions that link at least two upstream and two downstream diagrams or diagram groups. To calculate new upstream regulators of existing diagrams, we merged the EMMAA‐OP interactions with the network of existing crosstalk. We kept interactions with source elements, not within existing diagrams, and target elements in at least one existing diagram or diagram group.

#### Diagram interoperability and translation for computational modelling

Bidirectional translation of curated diagrams between CellDesigner and SBGNML formats is supported by the MINERVA Conversion API https://minerva.pages.uni.lu/doc/api/15.1/converter/ (Hoksza *et al*, 2020), and cd2sbgml converter https://github.com/sbgn/cd2sbgnml (Balaur *et al*, 2020). The MINERVA Conversion API supports bidirectional translation between CellDesigner, SBML and GPML. Unidirectional translation from Reactome format to GPML is supported by the Reactome‐to‐WikiPathways converter (Bohler *et al*, 2016). Diagrams in the RNEF format of Elsevier Pathway Studio were translated to SBGNML using a dedicated rnef2sbgn software (https://github.com/golovatenkop/rnef2sbgn).

The C19DMap diagrams (Table EV1) in CellDesigner format were translated using CaSQ (Aghamiri *et al*, 2020) into executable Boolean networks. Conversion rules and logical formulae were inferred according to the topology and the annotations of the diagrams. SBML‐qual files (Chaouiya *et al*, 2013) generated with CaSQ (Aghamiri *et al*, 2020) retained their references, annotations and layout of the original CellDesigner file. They can be used for *in silico* simulations and analysis with CellCollective (Helikar *et al*, 2012), GINsim (Naldi *et al*, 2018a) or MaBoSS (Stoll *et al*, 2017). CaSQ was adapted to produce SIF files necessary for HiPATHIA (Hidalgo *et al*, 2017) and CARNIVAL (Liu *et al*, 2019) pipelines. The C19DMap GitLab repository (https://gitlab.lcsb.uni.lu/covid/models) was configured to translate stable versions of diagrams into SBML qual and SIF files. The diagrams were translated to XGMML using Cytoscape and GINSim.

#### Calculation and visualisation of single‐cell RNA‐Seq expression profiles

RNA‐Seq transcriptomic single‐cell profiles were calculated for (i) non‐infected airway cells (Lukassen *et al*, 2020; Data ref: Lukassen *et al*, 2020) and (ii) SARS‐CoV‐2‐infected intestinal organoids (Triana *et al*, 2021; Data ref: Triana *et al*, 2021). The Seurat package (Hao *et al*, 2021) was used to calculate cell‐specific transcriptional profiles. For dataset (i), differential expression was calculated using every cell type against remaining cell types and applying the *FindAllMarkers* function of the Seurat package with min pct 0.25 and log fold change threshold 0.25. For dataset (ii), the cells were classified into bystander or infected based on the absence or presence of SARS‐CoV‐2 mRNA measured by scRNAseq (Triana *et al*, 2021; Data ref: Triana *et al*, 2021). Differential expression was calculated by contrasting the mock organoids with the bystander or infected cells after 12 h or 24 h of treatment. Expression profiles for the following cell types and conditions were selected for visualisation and enrichment analysis: for dataset (i), three types of secretory cells; and for dataset (ii), infected and bystander immature enterocytes 24 h post‐infection versus mock. The datasets were selected to recapitulate the findings in the original papers and demonstrate the capability of the C19DMap for cell‐specific data interpretation. Selected differentially expressed genes (DEGs) were prepared for visualisation in the MINERVA Platform as follows. Differential expression values were normalised to [−1,1] range by dividing by three and setting the outliers to their respective border values. Expression values and their corresponding HGNC symbols were used to create visual overlays in the C19DMap in the MINERVA Platform (https://covid19map.elixir‐luxembourg.org/minerva/index.xhtml?id=covid19_map_17Jun21, “Overlays” tab). On‐the‐fly exploration and enrichment analyses using the GSEA plugin (Hoksza *et al*, 2019) are described in a dedicated guide (https://covid.pages.uni.lu/minerva‐guide/). Complete expression analysis and transformation scripts are available in RMarkdown files at https://gitlab.lcsb.uni.lu/covid/models/‐/tree/master/Resources/Omics%20analysis.

#### RNA‐Seq‐based analysis of transcription factor activity

RNA‐Seq transcriptomic profiles of SARS‐CoV‐2 infection come from SARS‐CoV‐2‐infected Calu‐3 cells measured 24 h after infection, Gene Expression Omnibus reference GSE147507 (Blanco‐Melo *et al*, 2020; Data ref: Blanco‐Melo *et al*, 2020). Differential expression analysis of the transcript abundances between conditions was performed with DESeq2 (Love *et al*, 2014). The resulting *t*‐values from the differential expression analysis were used to estimate the effect of SARS‐CoV‐2 at the transcription factor (TF) activity level. This analysis was performed using the software Viper (Alvarez *et al*, 2016) algorithm coupled with TF–target interactions from DoRothEA (Garcia‐Alonso *et al*, 2019). DoRothEA TF–target interactions have a confidence level based on the reliability of their source, which ranges from A (most reliable) to E (least reliable). Here, interactions with confidence levels A, B and C were selected. Activities of TFs having at least five different targets were computed. The TFs normalised enrichment score from the Viper output was mapped on the “Interferon type I signalling pathway diagram” (https://fairdomhub.org/models/713) of the C19DMap using the SIF files generated by CaSQ. The resulting network was visualised using Cytoscape (Shannon *et al*, 2003). Notebooks to reproduce the results of this case study are available at https://github.com/saezlab/Covid19.

#### RNA‐seq‐based analysis of pathway signalling

The CoV‐HiPathia (Rian *et al*, 2021) web tool was used to calculate the level of activity of the subpathways of the apoptosis diagram (https://fairdomhub.org/models/712) from the C19DMap. RNA‐Seq transcriptomic profiles come from a public dataset of nasopharyngeal swabs from 430 individuals with SARS‐CoV‐2 and 54 negative controls, Gene Expression Omnibus reference GSE152075 (Lieberman *et al*, 2020; Data ref: Lieberman *et al*, 2020). RNA‐Seq gene expression data with the trimmed mean of M‐values (TMM) normalisation (Robinson *et al*, 2010) were rescaled to the range [0;1] for the calculation of the signal and normalised using quantile normalisation (Bolstad *et al*, 2003). Normalised gene expression values and the experimental design (case/control sample names files) were uploaded to CoV‐Hipathia to calculate the level of activation of the signalling in the selected diagram. A case/control contrast with a Wilcoxon test was used to assess differences in signalling activity between the two conditions. To reproduce the results, files with normalised gene expression data and the experimental design can be generated using the code https://gitlab.lcsb.uni.lu/covid/models/‐/tree/master/Resources/Hipathia/data_preprocessing. These files can then be used in CoV‐HiPathia at http://hipathia.babelomics.org/covid19/ under the “Differential signalling” tab. Diagrams from the C19DMap can be selected in the “Pathway source” section, under “Disease Maps Community curated pathways”.

## Author contributions

MO, AN, AM and IK planned and coordinated the project. RP, AO‐R, J‐MR, RF, VO and SM advised the project as domain experts. MO, AN, AM, IK, VS, SSA, MLA, EG, AR, GF, CM, BB, GF, LCMG, JS, MH, SG, JS, HB, TC, FS, AM, MPL, AF, YH, NH, TGY, AD, AR, MN, ZB, FM, DB, LF, MC, MR, VN, JV, LS, MW, EEA, JS, JZ, KO, JT, EK, GYS, KH, MK, SC, LE, FE, DABR, DS, MM, NP, RH, BJ, LM, MO‐M, ASR, KR, VS, RS, CS and TV curated and reviewed the diagrams. MO, PG, ES, LH, VS, GW, AR, MG, SO, CG and XH designed, developed and implemented key elements of the data sharing and communication infrastructure. RW. Overall, DM, AB, BMG, JAB, CV, VG, MV, PP, LL, MI, FS, AN, AY and AW designed and developed the contents of interaction and pathway databases, and text mining platforms and their visualisation and interoperability functionalities. AN, DT, AL, OB, SS, AV, ME, MP, KR, TH, BLP, DM, AT, MO, BDM, SB, AD, AN, VN and LC developed format interoperability, analysis and modelling workflows. CS, ED, TK, TF, FA, JSB, JH, OW, ELW, ARP, CTE, MEG, LS, HH, PD'E, JS‐R, JD, AV, HK, EB, CA, RB and RS defined the strategy and scope of the project and revised its progress. MO, AN, AM and IK wrote the manuscript. AO‐R, IK and AM designed the overview figures. AW, PD’E, JSB and LDS revised and contributed significantly to the structure of the manuscript. All authors have revised, read and accepted the manuscript in its final form.

## Conflict of interest

A. Niarakis collaborates with SANOFI‐AVENTIS R&D via a public–private partnership grant (CIFRE contract, n° 2020/0766). D. Maier and A. Bauch are employed at Biomax Informatics AG and will be affected by any effect of this publication on the commercial version of the AILANI software. J.A. Bachman and B. Gyori received consulting fees from Two Six Labs, LLC. T. Helikar has served as a shareholder and/or has consulted for Discovery Collective, Inc. R. Balling and R. Schneider are founders and shareholders of MEGENO S.A. and ITTM S.A. J. Saez‐Rodriguez receives funding from GSK and Sanofi and consultant fees from Travere Therapeutics. The remaining authors have declared that they have no Conflict of interest.

## Supporting information



Expanded View Figures PDF

Table EV1

Table EV2

## Data Availability

COVID‐19 Disease Map diagrams are available via:
‐ the GitLab repository (https://gitlab.lcsb.uni.lu/covid/models). ‐ WikiPathways collection (http://covid.wikipathways.org). ‐ Reactome collection (https://reactome.org/PathwayBrowser/#/R‐HSA‐9679506). Workflows, executable models and network models are available via:
‐ the GitLab repository (https://gitlab.lcsb.uni.lu/covid/models). ‐ FAIRDOMHub (https://fairdomhub.org/projects/190).

## References

[msb202110387-bib-0001] Aghamiri SS , Singh V , Naldi A , Helikar T , Soliman S , Niarakis A (2020) Automated inference of Boolean models from molecular interaction maps using CaSQ. Bioinforma Oxf Engl 36: 4473–4482 10.1093/bioinformatics/btaa484 PMC7575051 32403123

[msb202110387-bib-0002] Alvarez MJ , Shen Y , Giorgi FM , Lachmann A , Ding BB , Ye BH , Califano A (2016) Functional characterization of somatic mutations in cancer using network‐based inference of protein activity. Nat Genet 48: 838–847 27322546 10.1038/ng.3593 PMC5040167

[msb202110387-bib-0003] Amraei R , Yin W , Napoleon MA , Suder EL , Berrigan J , Zhao Q , Olejnik J , Chandler KB , Xia C , Feldman J *et al* (2020) CD209L/L‐SIGN and CD209/DC‐SIGN act as receptors for SARS‐CoV‐2 and are differentially expressed in lung and kidney epithelial and endothelial cells. BioRxiv 10.1101/2020.06.22.165803 [PREPRINT] PMC8265543 34341769

[msb202110387-bib-0004] Angelini MM , Akhlaghpour M , Neuman BW , Buchmeier MJ (2013) Severe acute respiratory syndrome coronavirus nonstructural proteins 3, 4, and 6 induce double‐membrane vesicles. Mbio 4: e00524‐13 10.1128/mBio.00524-13 PMC3747587 23943763

[msb202110387-bib-0005] Attucks OC , Jasmer KJ , Hannink M , Kassis J , Zhong Z , Gupta S , Victory SF , Guzel M , Polisetti DR , Andrews R *et al* (2014) Induction of heme oxygenase I (HMOX1) by HPP‐4382: a novel modulator of Bach1 activity. PLoS One 9: e101044 25019514 10.1371/journal.pone.0101044 PMC4096395

[msb202110387-bib-0006] Balaur I , Roy L , Mazein A , Karaca SG , Dogrusoz U , Barillot E , Zinovyev A (2020) cd2sbgnml: bidirectional conversion between cell designer and SBGN formats. Bioinform Oxf Engl 36: 2620–2622 10.1093/bioinformatics/btz969 31904823

[msb202110387-bib-0007] Bao C , Liu X , Zhang H , Li Y , Liu J (2020) Coronavirus disease 2019 (COVID‐19) CT findings: A Systematic Review and Meta‐analysis. J Am Coll Radiol JACR 17: 701–709 32283052 10.1016/j.jacr.2020.03.006 PMC7151282

[msb202110387-bib-0008] Bastard P , Rosen LB , Zhang Q , Michailidis E , Hoffmann H‐H , Zhang Y , Dorgham K , Philippot Q , Rosain J , Béziat V *et al* (2020) Auto‐antibodies against type I IFNs in patients with life‐threatening COVID‐19. Science 370: eabd4585 32972996 10.1126/science.abd4585 PMC7857397

[msb202110387-bib-0009] Batra N , De Souza C , Batra J , Raetz AG , Yu A‐M (2020) The HMOX1 pathway as a promising target for the treatment and prevention of SARS‐CoV‐2 of 2019 (COVID‐19). Int J Mol Sci 21: 6412 10.3390/ijms21176412 PMC7503392 32899231

[msb202110387-bib-0010] Bauch A , Pellet J , Schleicher T , Yu X , Gelemanović A , Cristella C , Fraaij PL , Polasek O , Auffray C , Maier D *et al* (2020) Informing epidemic (research) responses in a timely fashion by knowledge management ‐ a Zika virus use case. Biol Open 9: bio053934 33148605 10.1242/bio.053934 PMC7725600

[msb202110387-bib-0011] Bello‐Perez M , Sola I , Novoa B , Klionsky DJ , Falco A (2020) Canonical and noncanonical autophagy as potential targets for COVID‐19. Cells 9: 1619 10.3390/cells9071619 PMC7408018 32635598

[msb202110387-bib-0012] Bergmann FT , Czauderna T , Dogrusoz U , Rougny A , Dräger A , Touré V , Mazein A , Blinov ML , Luna A (2020) Systems biology graphical notation markup language (SBGNML) version 0.3. J Integr Bioinforma 17: 20200016 10.1515/jib-2020-0016 PMC7756621 32568733

[msb202110387-bib-0013] Bergmann FT , Keating SM , Gauges R , Sahle S , Wengler K (2018) SBML level 3 package: Render, Version 1, Release 1. J Integr Bioinforma 15: 20170078 10.1515/jib-2017-0078 PMC6167038 29605822

[msb202110387-bib-0014] Berthelot J‐M , Lioté F (2020) COVID‐19 as a STING disorder with delayed over‐secretion of interferon‐beta. EBioMedicine 56: 102801 32454408 10.1016/j.ebiom.2020.102801 PMC7244443

[msb202110387-bib-0015] Blanco‐Melo D , Nilsson‐Payant BE , Liu W‐C , Uhl S , Hoagland D , Møller R , Jordan TX , Oishi K , Panis M , Sachs D *et al* (2020) imbalanced host response to SARS‐CoV‐2 drives development of COVID‐19. Cell 181: 1036–1045 32416070 10.1016/j.cell.2020.04.026 PMC7227586

[msb202110387-bib-0016] Blanco‐Melo D , Nilsson‐Payant BE , Liu W‐C , Uhl S , Hoagland D , Møller R , Jordan TX , Oishi K , Panis M , Sachs D *et al* (2020) Gene Expression Omnibus GSE147507 (https://www.ncbi.nlm.nih.gov/geo/query/acc.cgi?acc=GSE147507). [DATASET]

[msb202110387-bib-0017] Bohler A , Wu G , Kutmon M , Pradhana LA , Coort SL , Hanspers K , Haw R , Pico AR , Evelo CT (2016) Reactome from a WikiPathways perspective. PLoS Comput Biol 12: e1004941 27203685 10.1371/journal.pcbi.1004941 PMC4874630

[msb202110387-bib-0018] Bolstad BM , Irizarry RA , Astrand M , Speed TP (2003) A comparison of normalization methods for high density oligonucleotide array data based on variance and bias. Bioinforma Oxf Engl 19: 185–193 10.1093/bioinformatics/19.2.185 12538238

[msb202110387-bib-0019] Bouhaddou M , Memon D , Meyer B , White KM , Rezelj VV , Correa Marrero M , Polacco BJ , Melnyk JE , Ulferts S , Kaake RM *et al* (2020) The global phosphorylation landscape of SARS‐CoV‐2 infection. Cell 182: 685–712 32645325 10.1016/j.cell.2020.06.034 PMC7321036

[msb202110387-bib-0020] Chaouiya C , Bérenguier D , Keating SM , Naldi A , van Iersel MP , Rodriguez N , Dräger A , Büchel F , Cokelaer T , Kowal B *et al* (2013) SBML qualitative models: a model representation format and infrastructure to foster interactions between qualitative modelling formalisms and tools. BMC Syst Biol 7: 135 24321545 10.1186/1752-0509-7-135 PMC3892043

[msb202110387-bib-0320] Chauhan D , Bartok E , Gaidt MM , Bock FJ , Herrmann J , Seeger JM , Broz P , Beckmann R , Kashkar H , Tait SWG *et al* (2018) BAX/BAK‐induced apoptosis results in caspase‐8‐dependent IL‐1β maturation in macrophages. Cell Rep 25: 2354–2368.e5 30485805 10.1016/j.celrep.2018.10.087

[msb202110387-bib-0021] Chen G , Wu DI , Guo W , Cao Y , Huang DA , Wang H , Wang T , Zhang X , Chen H , Yu H *et al* (2020a) Clinical and immunological features of severe and moderate coronavirus disease 2019. J Clin Invest 130: 2620–2629 32217835 10.1172/JCI137244 PMC7190990

[msb202110387-bib-0022] Chen S , Jonas F , Shen C , Hilgenfeld R , Higenfeld R (2010) Liberation of SARS‐CoV main protease from the viral polyprotein: N‐terminal autocleavage does not depend on the mature dimerization mode. Protein Cell 1: 59–74 21203998 10.1007/s13238-010-0011-4 PMC4875104

[msb202110387-bib-0023] Chen X , Yang X , Zheng Y , Yang Y , Xing Y , Chen Z (2014) SARS coronavirus papain‐like protease inhibits the type I interferon signaling pathway through interaction with the STING‐TRAF3‐TBK1 complex. Protein Cell 5: 369–381 24622840 10.1007/s13238-014-0026-3 PMC3996160

[msb202110387-bib-0024] Chen Y , Feng Z , Diao B , Wang R , Wang G , Wang C , Tan Y , Liu L , Wang C , Liu Y *et al* (2020b) The novel severe acute respiratory syndrome coronavirus 2 (SARS‐CoV‐2) directly decimates human spleens and lymph nodes. medRxiv 10.1101/2020.03.27.20045427 [PREPRINT]

[msb202110387-bib-0025] Cheon H , Holvey‐Bates EG , Schoggins JW , Forster S , Hertzog P , Imanaka N , Rice CM , Jackson MW , Junk DJ , Stark GR (2013) IFNβ‐dependent increases in STAT1, STAT2, and IRF9 mediate resistance to viruses and DNA damage. EMBO J 32: 2751–2763 24065129 10.1038/emboj.2013.203 PMC3801437

[msb202110387-bib-0026] Choi Y , Bowman JW , Jung JU (2018) Autophagy during viral infection – a double‐edged sword. Nat Rev Microbiol 16: 341–354 29556036 10.1038/s41579-018-0003-6 PMC6907743

[msb202110387-bib-0027] Chu H , Chan JF‐W , Wang Y , Yuen TT‐T , Chai Y , Hou Y , Shuai H , Yang D , Hu B , Huang X *et al* (2020) Comparative replication and immune activation profiles of SARS‐CoV‐2 and SARS‐CoV in human lungs: an ex vivo study with implications for the pathogenesis of COVID‐19. Clin Infect Dis Publ Infect Dis Soc Am 71: 1400–1409 10.1093/cid/ciaa410 PMC7184390 32270184

[msb202110387-bib-0028] Chu H , Zhou J , Wong BH‐Y , Li C , Chan JF‐W , Cheng Z‐S , Yang D , Wang D , Lee AC‐Y , Li C *et al* (2016) Middle east respiratory syndrome coronavirus efficiently infects human primary T lymphocytes and activates the extrinsic and intrinsic apoptosis pathways. J Infect Dis 213: 904–914 26203058 10.1093/infdis/jiv380 PMC7107330

[msb202110387-bib-0029] Courtot M , Juty N , Knüpfer C , Waltemath D , Zhukova A , Dräger A , Dumontier M , Finney A , Golebiewski M , Hastings J *et al* (2011) Controlled vocabularies and semantics in systems biology. Mol Syst Biol 7: 543 22027554 10.1038/msb.2011.77 PMC3261705

[msb202110387-bib-0030] Czauderna T , Klukas C , Schreiber F (2010) Editing, validating and translating of SBGN maps. Bioinforma Oxf Engl 26: 2340–2341 10.1093/bioinformatics/btq407 PMC2935428 20628075

[msb202110387-bib-0031] Daraselia N , Yuryev A , Egorov S , Novichkova S , Nikitin A , Mazo I (2004) Extracting human protein interactions from MEDLINE using a full‐sentence parser. Bioinforma Oxf Engl 20: 604–611 10.1093/bioinformatics/btg452 15033866

[msb202110387-bib-0032] De Meulder B , Lefaudeux D , Bansal AT , Mazein A , Chaiboonchoe A , Ahmed H , Balaur I , Saqi M , Pellet J , Ballereau S *et al* (2018) A computational framework for complex disease stratification from multiple large‐scale datasets. BMC Syst Biol 12: 60 29843806 10.1186/s12918-018-0556-z PMC5975674

[msb202110387-bib-0033] DeDiego ML , Nieto‐Torres JL , Jiménez‐Guardeño JM , Regla‐Nava JA , Alvarez E , Oliveros JC , Zhao J , Fett C , Perlman S , Enjuanes L (2011) Severe acute respiratory syndrome coronavirus envelope protein regulates cell stress response and apoptosis. PLoS Pathog 7: e1002315 22028656 10.1371/journal.ppat.1002315 PMC3197621

[msb202110387-bib-0034] Delorey TM , Ziegler CGK , Heimberg G , Normand R , Yang Y , Segerstolpe Å , Abbondanza D , Fleming SJ , Subramanian A , Montoro DT *et al* (2021) COVID‐19 tissue atlases reveal SARS‐CoV‐2 pathology and cellular targets. Nature 595: 107–113 33915569 10.1038/s41586-021-03570-8 PMC8919505

[msb202110387-bib-0035] Demir E , Cary MP , Paley S , Fukuda K , Lemer C , Vastrik I , Wu G , D’Eustachio P , Schaefer C , Luciano J *et al* (2010) The BioPAX community standard for pathway data sharing. Nat Biotechnol 28: 935–942 20829833 10.1038/nbt.1666 PMC3001121

[msb202110387-bib-0036] Devaraj SG , Wang N , Chen Z , Chen Z , Tseng M , Barretto N , Lin R , Peters CJ , Tseng C‐TK , Baker SC *et al* (2007) Regulation of IRF‐3‐dependent innate immunity by the papain‐like protease domain of the severe acute respiratory syndrome coronavirus. J Biol Chem 282: 32208–32221 17761676 10.1074/jbc.M704870200 PMC2756044

[msb202110387-bib-0037] Diemer C , Schneider M , Schätzl HM , Gilch S (2010) Modulation of host cell death by SARS coronavirus proteins. In Molecular biology of the SARS‐coronavirus, Lal SK (ed) pp 231–245. Berlin, Heidelberg: Springer Berlin Heidelberg

[msb202110387-bib-0038] Dugourd A , Kuppe C , Sciacovelli M , Gjerga E , Gabor A , Emdal KB , Vieira V , Bekker‐Jensen DB , Kranz J , EricMJ B *et al* (2021) Causal integration of multi‐omics data with prior knowledge to generate mechanistic hypotheses. Mol Syst Biol 17: e9730 33502086 10.15252/msb.20209730 PMC7838823

[msb202110387-bib-0039] Dugourd A , Saez‐Rodriguez J (2019) Footprint‐based functional analysis of multiomic data. Curr Opin Syst Biol 15: 82–90 32685770 10.1016/j.coisb.2019.04.002 PMC7357600

[msb202110387-bib-0040] Fang R , Wang C , Jiang Q , Lv M , Gao P , Yu X , Mu P , Zhang R , Bi S , Feng J‐M *et al* (2017) (2017) NEMO‐IKKβ are essential for IRF3 and NF‐κB activation in the cGAS‐STING pathway. J Immunol Baltim Md 199: 3222–3233 10.4049/jimmunol.1700699 28939760

[msb202110387-bib-0041] Fink K , Grandvaux N (2013) STAT2 and IRF9: Beyond ISGF3. JAK‐STAT 2: e27521 24498542 10.4161/jkst.27521 PMC3906322

[msb202110387-bib-0042] Frieman M , Yount B , Heise M , Kopecky‐Bromberg SA , Palese P , Baric RS (2007) Severe acute respiratory syndrome coronavirus ORF6 antagonizes STAT1 function by sequestering nuclear import factors on the rough endoplasmic reticulum/Golgi membrane. J Virol 81: 9812–9824 17596301 10.1128/JVI.01012-07 PMC2045396

[msb202110387-bib-0043] Fukushi M , Yoshinaka Y , Matsuoka Y , Hatakeyama S , Ishizaka Y , Kirikae T , Sasazuki T , Miyoshi‐Akiyama T (2012) Monitoring of S protein maturation in the endoplasmic reticulum by calnexin is important for the infectivity of severe acute respiratory syndrome coronavirus. J Virol 86: 11745–11753 22915798 10.1128/JVI.01250-12 PMC3486308

[msb202110387-bib-0044] Fung TS , Liu DX (2019) Human coronavirus: host‐pathogen interaction. Annu Rev Microbiol 73: 529–557 31226023 10.1146/annurev-micro-020518-115759

[msb202110387-bib-0045] Gagliardi I , Patella G , Michael A , Serra R , Provenzano M , Andreucci M (2020) COVID‐19 and the kidney: from epidemiology to clinical practice. J Clin Med 9: 2506 10.3390/jcm9082506 PMC7464116 32759645

[msb202110387-bib-0046] Gao C , Zeng J , Jia N , Stavenhagen K , Matsumoto Y , Zhang H , Li J , Hume AJ , Mühlberger E , van Die I *et al* (2020) SARS‐CoV‐2 Spike Protein Interacts with Multiple Innate Immune Receptors. BioRxiv 10.1101/2020.07.29.227462 [PREPRINT]

[msb202110387-bib-0047] Garcia‐Alonso L , Holland CH , Ibrahim MM , Turei D , Saez‐Rodriguez J (2019) Benchmark and integration of resources for the estimation of human transcription factor activities. Genome Res 29: 1363–1375 31340985 10.1101/gr.240663.118 PMC6673718

[msb202110387-bib-0048] Gauges R , Rost U , Sahle S , Wengler K , Bergmann FT (2015) The Systems Biology Markup Language (SBML) Level 3 Package: Layout, Version 1 Core. J Integr Bioinforma 12: 267 10.2390/biecoll-jib-2015-267 26528565

[msb202110387-bib-0049] Gawron P , Ostaszewski M , Satagopam V , Gebel S , Mazein A , Kuzma M , Zorzan S , McGee F , Otjacques B , Balling R *et al* (2016) MINERVA‐a platform for visualization and curation of molecular interaction networks. NPJ Syst Biol Appl 2: 16020 28725475 10.1038/npjsba.2016.20 PMC5516855

[msb202110387-bib-0050] Ghaffarizadeh A , Heiland R , Friedman SH , Mumenthaler SM , Macklin P (2018) PhysiCell: an open source physics‐based cell simulator for 3‐D multicellular systems. PLoS Comput Biol 14: e1005991 29474446 10.1371/journal.pcbi.1005991 PMC5841829

[msb202110387-bib-0051] Gheblawi M , Wang K , Viveiros A , Nguyen Q , Zhong J‐C , Turner AJ , Raizada MK , Grant MB , Oudit GY (2020) Angiotensin‐Converting enzyme 2: SARS‐CoV‐2 receptor and regulator of the renin‐angiotensin system: celebrating the 20th anniversary of the discovery of ACE2. Circ Res 126: 1456–1474 32264791 10.1161/CIRCRESAHA.120.317015 PMC7188049

[msb202110387-bib-0052] Ghosh S , Dellibovi‐Ragheb TA , Kerviel A , Pak E , Qiu Q , Fisher M , Takvorian PM , Bleck C , Hsu VW , Fehr AR *et al* (2020) β‐Coronaviruses use lysosomes for egress instead of the biosynthetic secretory pathway. Cell 183: 1520–1535 33157038 10.1016/j.cell.2020.10.039 PMC7590812

[msb202110387-bib-0053] Gordon DE , Jang GM , Bouhaddou M , Xu J , Obernier K , White KM , O’Meara MJ , Rezelj VV , Guo JZ , Swaney DL *et al* (2020) A SARS‐CoV‐2 protein interaction map reveals targets for drug repurposing. Nature 583: 459–468 32353859 10.1038/s41586-020-2286-9 PMC7431030

[msb202110387-bib-0055] Gyori BM , Bachman JA , Subramanian K , Muhlich JL , Galescu L , Sorger PK (2017) From word models to executable models of signaling networks using automated assembly. Mol Syst Biol 13: 954 29175850 10.15252/msb.20177651 PMC5731347

[msb202110387-bib-0056] Häcker H , Karin M (2006) Regulation and function of IKK and IKK‐related kinases. Sci STKE Signal Transduct Knowl Environ 2006: re13 10.1126/stke.3572006re13 17047224

[msb202110387-bib-0057] Hadjadj J , Yatim N , Barnabei L , Corneau A , Boussier J , Smith N , Péré H , Charbit B , Bondet V , Chenevier‐Gobeaux C *et al* (2020) Impaired type I interferon activity and inflammatory responses in severe COVID‐19 patients. Science 369: 718–724 32661059 10.1126/science.abc6027 PMC7402632

[msb202110387-bib-0058] Haga S , Yamamoto N , Nakai‐Murakami C , Osawa Y , Tokunaga K , Sata T , Yamamoto N , Sasazuki T , Ishizaka Y (2008) Modulation of TNF‐alpha‐converting enzyme by the spike protein of SARS‐CoV and ACE2 induces TNF‐alpha production and facilitates viral entry. Proc Natl Acad Sci USA 105: 7809–7814 18490652 10.1073/pnas.0711241105 PMC2409424

[msb202110387-bib-0059] Hanspers K , Riutta A , Summer‐Kutmon M , Pico AR (2020) Pathway information extracted from 25 years of pathway figures. Genome Biol 21:273 33168034 10.1186/s13059-020-02181-2 PMC7649569

[msb202110387-bib-0060] Hao Y , Hao S , Andersen‐Nissen E , Mauck WM , Zheng S , Butler A , Lee MJ , Wilk AJ , Darby C , Zager M *et al* (2021) Integrated analysis of multimodal single‐cell data. Cell 184: 3573–3587 34062119 10.1016/j.cell.2021.04.048 PMC8238499

[msb202110387-bib-0061] Hassan SM , Jawad MJ , Ahjel SW , Singh RB , Singh J , Awad SM , Hadi NR (2020) The Nrf2 activator (DMF) and covid‐19: is there a possible role? Med Arch Sarajevo Bosnia Herzeg 74: 134–138 10.5455/medarh.2020.74.134-138 PMC7296400 32577056

[msb202110387-bib-0062] Hayek S , Pietrancosta N , Hovhannisyan AA , Alves de Sousa R , Bekaddour N , Ermellino L , Tramontano E , Arnould S , Sardet C , Dairou J *et al* (2020) Cerpegin‐derived furo[3,4‐c]pyridine‐3,4(1H,5H)‐diones enhance cellular response to interferons by de novo pyrimidine biosynthesis inhibition. Eur J Med Chem 186: 111855 31740051 10.1016/j.ejmech.2019.111855

[msb202110387-bib-0063] Helikar T , Kowal B , McClenathan S , Bruckner M , Rowley T , Madrahimov A , Wicks B , Shrestha M , Limbu K , Rogers JA (2012) The Cell Collective: Toward an open and collaborative approach to systems biology. BMC Syst Biol 6: 96 22871178 10.1186/1752-0509-6-96 PMC3443426

[msb202110387-bib-0064] Hidalgo MR , Cubuk C , Amadoz A , Salavert F , Carbonell‐Caballero J , Dopazo J (2017) High throughput estimation of functional cell activities reveals disease mechanisms and predicts relevant clinical outcomes. Oncotarget 8: 5160–5178 28042959 10.18632/oncotarget.14107 PMC5354899

[msb202110387-bib-0065] Hoffmann M , Kleine‐Weber H , Pöhlmann S (2020a) A multibasic cleavage site in the spike protein of SARS‐CoV‐2 is essential for infection of human lung cells. Mol Cell 78: 779–784 32362314 10.1016/j.molcel.2020.04.022 PMC7194065

[msb202110387-bib-0066] Hoffmann M , Kleine‐Weber H , Schroeder S , Krüger N , Herrler T , Erichsen S , Schiergens TS , Herrler G , Wu N‐H , Nitsche A *et al* (2020b) SARS‐CoV‐2 cell entry depends on ACE2 and TMPRSS2 and is blocked by a clinically proven protease inhibitor. Cell 181: 271–280 32142651 10.1016/j.cell.2020.02.052 PMC7102627

[msb202110387-bib-0067] Hoksza D , Gawron P , Ostaszewski M , Smula E , Schneider R (2019) MINERVA API and plugins: opening molecular network analysis and visualization to the community. Bioinforma Oxf Engl 35: 4496–4498 10.1093/bioinformatics/btz286 PMC6821317 31074494

[msb202110387-bib-0068] Hoksza D , Gawron P , Ostaszewski M , Hasenauer J , Schneider R (2020) Closing the gap between formats for storing layout information in systems biology. Brief Bioinform 21: 1249–1260 31273380 10.1093/bib/bbz067 PMC7373180

[msb202110387-bib-0069] Huang C , Wang Y , Li X , Ren L , Zhao J , Hu YI , Zhang LI , Fan G , Xu J , Gu X *et al* (2020) Clinical features of patients infected with 2019 novel coronavirus in Wuhan, China. Lancet Lond Engl 395: 497–506 10.1016/S0140-6736(20)30183-5 PMC7159299 31986264

[msb202110387-bib-0070] Hui KPY , Cheung M‐C , Perera RAPM , Ng K‐C , Bui CHT , Ho JCW , Ng MMT , Kuok DIT , Shih KC , Tsao S‐W *et al* (2020) Tropism, replication competence, and innate immune responses of the coronavirus SARS‐CoV‐2 in human respiratory tract and conjunctiva: an analysis in ex‐vivo and in‐vitro cultures. Lancet Respir Med 8: 687–695 32386571 10.1016/S2213-2600(20)30193-4 PMC7252187

[msb202110387-bib-0071] Iba T , Levy JH , Connors JM , Warkentin TE , Thachil J , Levi M (2020) The unique characteristics of COVID‐19 coagulopathy. Crit Care Lond Engl 24: 360 10.1186/s13054-020-03077-0 PMC7301352 32552865

[msb202110387-bib-0072] van Iersel MP , Pico AR , Kelder T , Gao J , Ho I , Hanspers K , Conklin BR , Evelo CT (2010) The BridgeDb framework: standardized access to gene, protein and metabolite identifier mapping services. BMC Bioinformatics 11: 5 20047655 10.1186/1471-2105-11-5 PMC2824678

[msb202110387-bib-0073] Jassal B , Matthews L , Viteri G , Gong C , Lorente P , Fabregat A , Sidiropoulos K , Cook J , Gillespie M , Haw R *et al* (2020) The Reactome Pathway Knowledgebase. Nucleic Acids Res 48: D498–D503 31691815 10.1093/nar/gkz1031 PMC7145712

[msb202110387-bib-0074] Juty N , Le Novère N , Laibe C (2012) Identifiers.org and MIRIAM Registry: community resources to provide persistent identification. Nucleic Acids Res 40: D580–586 22140103 10.1093/nar/gkr1097 PMC3245029

[msb202110387-bib-0075] Kanzawa N , Nishigaki K , Hayashi T , Ishii Y , Furukawa S , Niiro A , Yasui F , Kohara M , Morita K , Matsushima K *et al* (2006) Augmentation of chemokine production by severe acute respiratory syndrome coronavirus 3a/X1 and 7a/X4 proteins through NF‐kappaB activation. FEBS Lett 580: 6807–6812 17141229 10.1016/j.febslet.2006.11.046 PMC7094718

[msb202110387-bib-0076] Keating SM , Waltemath D , König M , Zhang F , Dräger A , Chaouiya C , Bergmann FT , Finney A , Gillespie CS , Helikar T *et al* (2020) SBML Level 3: an extensible format for the exchange and reuse of biological models. Mol Syst Biol 16: e9110 32845085 10.15252/msb.20199110 PMC8411907

[msb202110387-bib-0077] Kedia‐Mehta N , Finlay DK (2019) Competition for nutrients and its role in controlling immune responses. Nat Commun 10: 2123 31073180 10.1038/s41467-019-10015-4 PMC6509329

[msb202110387-bib-0078] Kesic MJ , Simmons SO , Bauer R , Jaspers I (2011) Nrf2 expression modifies influenza A entry and replication in nasal epithelial cells. Free Radic Biol Med 51: 444–453 21549835 10.1016/j.freeradbiomed.2011.04.027 PMC3135631

[msb202110387-bib-0079] Klok FA , Kruip M , van der Meer N , Arbous MS , Gommers D , Kant KM , Kaptein F , van Paassen J , Stals M , Huisman MV *et al* (2020) Incidence of thrombotic complications in critically ill ICU patients with COVID‐19. Thromb Res 191: 145–147 32291094 10.1016/j.thromres.2020.04.013 PMC7146714

[msb202110387-bib-0080] Krähling V , Stein DA , Spiegel M , Weber F , Mühlberger E (2009) Severe acute respiratory syndrome coronavirus triggers apoptosis via protein kinase R but is resistant to its antiviral activity. J Virol 83: 2298–2309 19109397 10.1128/JVI.01245-08 PMC2643707

[msb202110387-bib-0081] Kutmon M , van Iersel MP , Bohler A , Kelder T , Nunes N , Pico AR , Evelo CT (2015) PathVisio 3: an extendable pathway analysis toolbox. PLoS Comput Biol 11: e1004085 25706687 10.1371/journal.pcbi.1004085 PMC4338111

[msb202110387-bib-0082] Lee JS , Shin E‐C (2020) The type I interferon response in COVID‐19: implications for treatment. Nat Rev Immunol 20: 585–586 32788708 10.1038/s41577-020-00429-3 PMC8824445

[msb202110387-bib-0083] Letko M , Marzi A , Munster V (2020) Functional assessment of cell entry and receptor usage for SARS‐CoV‐2 and other lineage B betacoronaviruses. Nat Microbiol 5: 562–569 32094589 10.1038/s41564-020-0688-y PMC7095430

[msb202110387-bib-0084] Letort G , Montagud A , Stoll G , Heiland R , Barillot E , Macklin P , Zinovyev A , Calzone L (2019) PhysiBoSS: a multi‐scale agent‐based modelling framework integrating physical dimension and cell signalling. Bioinforma Oxf Engl 35: 1188–1196 10.1093/bioinformatics/bty766 PMC6449758 30169736

[msb202110387-bib-0086] Li S , Zhang Y , Guan Z , Li H , Ye M , Chen X , Shen J , Zhou Y , Shi Z‐L , Zhou P *et al* (2020) SARS‐CoV‐2 triggers inflammatory responses and cell death through caspase‐8 activation. Signal Transduct Target Ther 5: 235 33037188 10.1038/s41392-020-00334-0 PMC7545816

[msb202110387-bib-0085] Li S‐W , Wang C‐Y , Jou Y‐J , Huang S‐H , Hsiao L‐H , Wan L , Lin Y‐J , Kung S‐H , Lin C‐W (2016) SARS coronavirus papain‐like protease inhibits the TLR7 signaling pathway through removing Lys63‐linked polyubiquitination of TRAF3 and TRAF6. Int J Mol Sci 17: 678 10.3390/ijms17050678 PMC4881504 27164085

[msb202110387-bib-0087] Liao Q‐J , Ye L‐B , Timani KA , Zeng Y‐C , She Y‐L , Ye L , Wu Z‐H (2005) Activation of NF‐kappaB by the full‐length nucleocapsid protein of the SARS coronavirus. Acta Biochim Biophys Sin 37: 607–612 16143815 10.1111/j.1745-7270.2005.00082.x PMC7109668

[msb202110387-bib-0088] Licata L , Lo Surdo P , Iannuccelli M , Palma A , Micarelli E , Perfetto L , Peluso D , Calderone A , Castagnoli L , Cesareni G (2020) SIGNOR 2.0, the SIGnaling network open resource 2.0: 2019 update. Nucleic Acids Res 48: D504–D510 31665520 10.1093/nar/gkz949 PMC7145695

[msb202110387-bib-0089] Lieberman NAP , Peddu V , Xie H , Shrestha L , Huang M‐L , Mears MC , Cajimat MN , Bente DA , Shi P‐Y , Bovier F *et al* (2020) Gene Expression Omnibus GSE152075 (https://www.ncbi.nlm.nih.gov/geo/query/acc.cgi?acc=GSE152075). [DATASET]

[msb202110387-bib-0090] Lieberman NAP , Peddu V , Xie H , Shrestha L , Huang M‐L , Mears MC , Cajimat MN , Bente DA , Shi P‐Y , Bovier F *et al* (2020) In vivo antiviral host transcriptional response to SARS‐CoV‐2 by viral load, sex, and age. PLoS Biol 18: e3000849 32898168 10.1371/journal.pbio.3000849 PMC7478592

[msb202110387-bib-0091] Liu A , Trairatphisan P , Gjerga E , Didangelos A , Barratt J , Saez‐Rodriguez J (2019) From expression footprints to causal pathways: contextualizing large signaling networks with CARNIVAL. NPJ Syst Biol Appl 5: 40 31728204 10.1038/s41540-019-0118-z PMC6848167

[msb202110387-bib-0092] Liu M , Yang Y , Gu C , Yue Y , Wu KK , Wu J , Zhu Y (2007) Spike protein of SARS‐CoV stimulates cyclooxygenase‐2 expression via both calcium‐dependent and calcium‐independent protein kinase C pathways. FASEB J Publ Fed Am Soc Exp Biol 21: 1586–1596 10.1096/fj.06-6589com 17267381

[msb202110387-bib-0093] Love MI , Huber W , Anders S (2014) Moderated estimation of fold change and dispersion for RNA‐seq data with DESeq2. Genome Biol 15: 550 25516281 10.1186/s13059-014-0550-8 PMC4302049

[msb202110387-bib-0094] Lu Wang L , Lo K , Chandrasekhar Y , Reas R , Yang J , Eide D , Funk K , Kinney R , Liu Z , Merrill W *et al* (2020) CORD‐19: The Covid‐19 Open Research Dataset. ArXiv https://arxiv.org/abs/2004.10706v2 [PREPRINT]

[msb202110387-bib-0095] Lubin JH , Zardecki C , Dolan EM , Lu C , Shen Z , Dutta S , Westbrook JD , Hudson BP , Goodsell DS , Williams JK *et al* (2020) Evolution of the SARS‐CoV‐2 proteome in three dimensions (3D) during the first six months of the COVID‐19 pandemic. BioRxiv 10.1101/2020.12.01.406637 [PREPRINT] PMC8661935 34580920

[msb202110387-bib-0096] Lucas C , Wong P , Klein J , Castro TBR , Silva J , Sundaram M , Ellingson MK , Mao T , Oh JE , Israelow B *et al* (2020) Longitudinal analyses reveal immunological misfiring in severe COVID‐19. Nature 584: 463–469 32717743 10.1038/s41586-020-2588-y PMC7477538

[msb202110387-bib-0097] Lukassen S , Chua RL , Trefzer T , Kahn NC , Schneider MA , Muley T , Winter H , Meister M , Veith C , Boots AW *et al* (2020) Figshare 11981034 (https://doi.org/10.6084/m9.figshare.11981034.v1). [DATASET] PMC7232010 32246845

[msb202110387-bib-0098] Lukassen S , Chua RL , Trefzer T , Kahn NC , Schneider MA , Muley T , Winter H , Meister M , Veith C , Boots AW *et al* (2020) SARS‐CoV‐2 receptor ACE2 and TMPRSS2 are primarily expressed in bronchial transient secretory cells. EMBO J 39: e105114 32246845 10.15252/embj.20105114 PMC7232010

[msb202110387-bib-0099] Magro C , Mulvey JJ , Berlin D , Nuovo G , Salvatore S , Harp J , Baxter‐Stoltzfus A , Laurence J (2020) Complement associated microvascular injury and thrombosis in the pathogenesis of severe COVID‐19 infection: A report of five cases. Transl Res J Lab Clin Med 220: 1–13 10.1016/j.trsl.2020.04.007 PMC7158248 32299776

[msb202110387-bib-0100] Mantlo E , Bukreyeva N , Maruyama J , Paessler S , Huang C (2020) Antiviral activities of type I interferons to SARS‐CoV‐2 infection. Antiviral Res 179: 104811 32360182 10.1016/j.antiviral.2020.104811 PMC7188648

[msb202110387-bib-0101] Mason RJ (2020) Pathogenesis of COVID‐19 from a cell biology perspective. Eur Respir J 55: 2000607 32269085 10.1183/13993003.00607-2020 PMC7144260

[msb202110387-bib-0102] Matsuoka Y , Funahashi A , Ghosh S , Kitano H (2014) Modeling and simulation using Cell Designer. Methods Mol Biol Clifton NJ 1164: 121–145 10.1007/978-1-4939-0805-9_11 24927840

[msb202110387-bib-0103] Mazein A , Ostaszewski M , Kuperstein I , Watterson S , Le Novère N , Lefaudeux D , De Meulder B , Pellet J , Balaur I , Saqi M *et al* (2018) Systems medicine disease maps: community‐driven comprehensive representation of disease mechanisms. NPJ Syst Biol Appl 4: 21 29872544 10.1038/s41540-018-0059-y PMC5984630

[msb202110387-bib-0104] McFadyen JD , Stevens H , Peter K (2020) The emerging threat of (Micro)Thrombosis in COVID‐19 and Its therapeutic implications. Circ Res 127: 571–587 32586214 10.1161/CIRCRESAHA.120.317447 PMC7386875

[msb202110387-bib-0105] Meldal BHM , Bye‐A‐Jee H , Gajdos\ˇ L , Hammerová Z , Horácková A , Melicher F , Perfetto L , Pokorný D , Lopez MR , Türková A *et al* (2019) Complex Portal 2018: extended content and enhanced visualization tools for macromolecular complexes. Nucleic Acids Res 47: D550–D558 30357405 10.1093/nar/gky1001 PMC6323931

[msb202110387-bib-0106] Mesev EV , LeDesma RA , Ploss A (2019) Decoding type I and III interferon signalling during viral infection. Nat Microbiol 4: 914–924 30936491 10.1038/s41564-019-0421-x PMC6554024

[msb202110387-bib-0107] Messina F , Giombini E , Agrati C , Vairo F , Ascoli Bartoli T , Al Moghazi S , Piacentini M , Locatelli F , Kobinger G , Maeurer M *et al* (2020) COVID‐19: viral‐host interactome analyzed by network based‐approach model to study pathogenesis of SARS‐CoV‐2 infection. J Transl Med 18: 233 32522207 10.1186/s12967-020-02405-w PMC7286221

[msb202110387-bib-0108] Miao G , Zhao H , Li Y , Ji M , Chen Y , Shi Y , Bi Y , Wang P , Zhang H (2020) ORF3a of the COVID‐19 virus SARS‐CoV‐2 blocks HOPS complex‐mediated assembly of the SNARE complex required for autolysosome formation. Dev Cell 56: 427–442 33422265 10.1016/j.devcel.2020.12.010 PMC7832235

[msb202110387-bib-0109] Mogensen TH (2009) Pathogen recognition and inflammatory signaling in innate immune defenses. Clin Microbiol Rev 22: 240–273 19366914 10.1128/CMR.00046-08 PMC2668232

[msb202110387-bib-0110] Murakami Y , Hoshi M , Imamura Y , Arioka Y , Yamamoto Y , Saito K (2013) Remarkable role of indoleamine 2,3‐dioxygenase and tryptophan metabolites in infectious diseases: potential role in macrophage‐mediated inflammatory diseases. Mediators Inflamm 2013: 391984 23476103 10.1155/2013/391984 PMC3588179

[msb202110387-bib-0111] Naithani S , Gupta P , Preece J , Garg P , Fraser V , Padgitt‐Cobb LK , Martin M , Vining K , Jaiswal P (2019) Involving community in genes and pathway curation. Database 2019: bay146 10.1093/database/bay146 PMC6334007 30649295

[msb202110387-bib-0112] Nakagawa K , Lokugamage KG , Makino S (2016) Viral and cellular mRNA translation in coronavirus‐infected cells. Adv Virus Res 96: 165–192 27712623 10.1016/bs.aivir.2016.08.001 PMC5388242

[msb202110387-bib-0113] Naldi A , Hernandez C , Abou‐Jaoudé W , Monteiro PT , Chaouiya C , Thieffry D (2018a) Logical modeling and analysis of cellular regulatory networks with GINsim 3.0. Front Physiol 9: 646 29971008 10.3389/fphys.2018.00646 PMC6018412

[msb202110387-bib-0114] Naldi A , Hernandez C , Levy N , Stoll G , Monteiro PT , Chaouiya C , Helikar T , Zinovyev A , Calzone L , Cohen‐Boulakia S *et al* (2018b) The CoLoMoTo interactive notebook: accessible and reproducible computational analyses for qualitative biological networks. Front Physiol 9: 680 29971009 10.3389/fphys.2018.00680 PMC6018415

[msb202110387-bib-0054] Nesterova AP , Klimov EA , Zharkova M , Sozin S , Sobolev V , Ivanikova NV , Shkrob M , Yuryev A (2020) Guide and legend. In Disease Pathways, pp xxi–xxviii. Cambridge, MA: Elsevier

[msb202110387-bib-0115] Niarakis A , Kuiper M , Ostaszewski M , Malik Sheriff RS , Casals‐Casas C , Thieffry D , Freeman TC , Thomas P , Touré V , Noël V *et al* (2020) Setting the basis of best practices and standards for curation and annotation of logical models in biology‐highlights of the [BC]2 2019 CoLoMoTo/SysMod Workshop. Brief Bioinform 22: 1848–1859. 10.1093/bib/bbaa046 PMC7986594 32313939

[msb202110387-bib-0116] Novère NL , Hucka M , Mi H , Moodie S , Schreiber F , Sorokin A , Demir E , Wegner K , Aladjem MI , Wimalaratne SM *et al* (2009) The systems biology graphical notation. Nat Biotechnol 27: 735–741 19668183 10.1038/nbt.1558

[msb202110387-bib-0117] Orchard S , Kerrien S , Abbani S , Aranda B , Bhate J , Bidwell S , Bridge A , Briganti L , Brinkman FSL , Brinkman F *et al* (2012) Protein interaction data curation: the International Molecular Exchange (IMEx) consortium. Nat Methods 9: 345–350 22453911 10.1038/nmeth.1931 PMC3703241

[msb202110387-bib-0118] Osborne JM , Fletcher AG , Pitt‐Francis JM , Maini PK , Gavaghan DJ (2017) Comparing individual‐based approaches to modelling the self‐organization of multicellular tissues. PLoS Comput Biol 13: e1005387 28192427 10.1371/journal.pcbi.1005387 PMC5330541

[msb202110387-bib-0119] Ostaszewski M , Mazein A , Gillespie ME , Kuperstein I , Niarakis A , Hermjakob H , Pico AR , Willighagen EL , Evelo CT , Hasenauer J *et al* (2020) COVID‐19 Disease Map, building a computational repository of SARS‐CoV‐2 virus‐host interaction mechanisms. Sci Data 7: 136 32371892 10.1038/s41597-020-0477-8 PMC7200764

[msb202110387-bib-0120] Park A , Iwasaki A (2020) Type I and type III interferons‐induction, signaling, evasion, and application to combat COVID‐19. Cell Host Microbe 27: 870–878 32464097 10.1016/j.chom.2020.05.008 PMC7255347

[msb202110387-bib-0121] Perfetto L , Pastrello C , Del‐Toro N , Duesbury M , Iannuccelli M , Kotlyar M , Licata L , Meldal B , Panneerselvam K , Panni S *et al* (2020) The IMEx coronavirus interactome: an evolving map of Coronaviridae‐host molecular interactions. Database (Oxford) 2020: baaa096 33206959 10.1093/database/baaa096 PMC7673336

[msb202110387-bib-0122] Pillich RT , Chen J , Rynkov V , Welker D , Pratt D (2017) NDEx: a community resource for sharing and publishing of biological networks. Methods Mol Biol Clifton NJ 1558: 271–301 10.1007/978-1-4939-6783-4_13 28150243

[msb202110387-bib-0123] Rao M , Dodoo E , Zumla A , Maeurer M (2019) Immunometabolism and pulmonary infections: implications for protective immune responses and host‐directed therapies. Front Microbiol 10: 962 31134013 10.3389/fmicb.2019.00962 PMC6514247

[msb202110387-bib-0124] Renz A , Widerspick L , Dräger A (2020) FBA reveals guanylate kinase as a potential target for antiviral therapies against SARS‐CoV‐2. Bioinforma Oxf Engl 36: i813–i821 10.1093/bioinformatics/btaa813 PMC7773487 33381848

[msb202110387-bib-0125] Rian K , Esteban‐Medina M , Hidalgo MR , Çubuk C , Falco MM , Loucera C , Gunyel D , Ostaszewski M , Peña‐Chilet M , Dopazo J (2021) Mechanistic modeling of the SARS‐CoV‐2 disease map. BioData Min 14: 5 33478554 10.1186/s13040-021-00234-1 PMC7817765

[msb202110387-bib-0126] Robinson MD , McCarthy DJ , Smyth GK (2010) edgeR: a Bioconductor package for differential expression analysis of digital gene expression data. Bioinforma Oxf Engl 26: 139–140 10.1093/bioinformatics/btp616 PMC2796818 19910308

[msb202110387-bib-0127] Rodchenkov I , Babur O , Luna A , Aksoy BA , Wong JV , Fong D , Franz M , Siper MC , Cheung M , Wrana M *et al* (2020) Pathway Commons 2019 Update: integration, analysis and exploration of pathway data. Nucleic Acids Res 48: D489–D497 31647099 10.1093/nar/gkz946 PMC7145667

[msb202110387-bib-0128] Rubio D , Xu R‐H , Remakus S , Krouse TE , Truckenmiller ME , Thapa RJ , Balachandran S , Alcamí A , Norbury CC , Sigal LJ (2013) Crosstalk between the type 1 interferon and nuclear factor kappa B pathways confers resistance to a lethal virus infection. Cell Host Microbe 13: 701–710 23768494 10.1016/j.chom.2013.04.015 PMC3688842

[msb202110387-bib-0129] Sa Ribero M , Jouvenet N , Dreux M , Nisole S (2020) Interplay between SARS‐CoV‐2 and the type I interferon response. PLoS Pathog 16: e1008737 32726355 10.1371/journal.ppat.1008737 PMC7390284

[msb202110387-bib-0130] Salavert F , Hidago MR , Amadoz A , Çubuk C , Medina I , Crespo D , Carbonell‐Caballero J , Dopazo J (2016) Actionable pathways: interactive discovery of therapeutic targets using signaling pathway models. Nucleic Acids Res 44: W212–216 27137885 10.1093/nar/gkw369 PMC4987920

[msb202110387-bib-0131] Satagopam V , Gu W , Eifes S , Gawron P , Ostaszewski M , Gebel S , Barbosa‐Silva A , Balling R , Schneider R (2016) Integration and visualization of translational medicine data for better understanding of human diseases. Big Data 4: 97–108 27441714 10.1089/big.2015.0057 PMC4932659

[msb202110387-bib-0132] Seo GJ , Kim C , Shin W‐J , Sklan EH , Eoh H , Jung JU (2018) TRIM56‐mediated monoubiquitination of cGAS for cytosolic DNA sensing. Nat Commun 9: 613 29426904 10.1038/s41467-018-02936-3 PMC5807518

[msb202110387-bib-0133] Shannon P , Markiel A , Ozier O , Baliga NS , Wang JT , Ramage D , Amin N , Schwikowski B , Ideker T (2003) Cytoscape: a software environment for integrated models of biomolecular interaction networks. Genome Res 13: 2498–2504 14597658 10.1101/gr.1239303 PMC403769

[msb202110387-bib-0134] Slayden RA , Jackson M , Zucker J , Ramirez MV , Dawson CC , Crew R , Sampson NS , Thomas ST , Jamshidi N , Sisk P *et al* (2013) Updating and curating metabolic pathways of TB. Tuberc Edinb Scotl 93: 47–59 10.1016/j.tube.2012.11.001 PMC4121119 23375378

[msb202110387-bib-0135] Slenter DN , Kutmon M , Hanspers K , Riutta A , Windsor J , Nunes N , Mélius J , Cirillo E , Coort SL , Digles D *et al* (2018) WikiPathways: a multifaceted pathway database bridging metabolomics to other omics research. Nucleic Acids Res 46: D661–D667 29136241 10.1093/nar/gkx1064 PMC5753270

[msb202110387-bib-0136] Smith JA (2018) Regulation of cytokine production by the unfolded protein response; implications for infection and autoimmunity. Front Immunol 9: 422 29556237 10.3389/fimmu.2018.00422 PMC5844972

[msb202110387-bib-0137] Stoll G , Caron B , Viara E , Dugourd A , Zinovyev A , Naldi A , Kroemer G , Barillot E , Calzone L (2017) MaBoSS 2.0: an environment for stochastic Boolean modeling. Bioinforma Oxf Engl 33: 2226–2228 10.1093/bioinformatics/btx123 28881959

[msb202110387-bib-0138] Su S , Jiang S (2020) A suspicious role of interferon in the pathogenesis of SARS‐CoV‐2 by enhancing expression of ACE2. Signal Transduct Target Ther 5: 71 32435059 10.1038/s41392-020-0185-z PMC7239689

[msb202110387-bib-0139] Su Y , Chen D , Lausted C , Yuan D , Choi J , Dai C , Voillet V , Scherler K , Troisch P , Duvvuri VR *et al* (2020) Multiomic immunophenotyping of COVID‐19 patients reveals early infection trajectories immunology. bioRxiv 10.1101/2020.07.27.224063 [PREPRINT]

[msb202110387-bib-0140] Sureda A , Alizadeh J , Nabavi SF , Berindan‐Neagoe I , Cismaru CA , Jeandet P , Łos MJ , Clementi E , Nabavi SM , Ghavami S (2020) Endoplasmic reticulum as a potential therapeutic target for covid‐19 infection management? Eur J Pharmacol 882: 173288 32561291 10.1016/j.ejphar.2020.173288 PMC7297682

[msb202110387-bib-0141] Takeuchi O , Akira S (2010) Pattern recognition receptors and inflammation. Cell 140: 805–820 20303872 10.1016/j.cell.2010.01.022

[msb202110387-bib-0142] Thoms M , Buschauer R , Ameismeier M , Koepke L , Denk T , Hirschenberger M , Kratzat H , Hayn M , Mackens‐Kiani T , Cheng J *et al* (2020) Structural basis for translational shutdown and immune evasion by the Nsp1 protein of SARS‐CoV‐2. Science 369: 1249– 1255 32680882 10.1126/science.abc8665 PMC7402621

[msb202110387-bib-0143] Triana S , Metz‐Zumaran C , Ramirez C , Kee C , Doldan P , Shahraz M , Schraivogel D , Gschwind AR , Sharma AK , Steinmetz LM *et al* (2021) Single‐cell analyses reveal SARS‐CoV‐2 interference with intrinsic immune response in the human gut. Mol Syst Biol 17: e10232 33904651 10.15252/msb.202110232 PMC8077299

[msb202110387-bib-0144] Triana S , Metz‐Zumaran C , Ramirez C , Kee C , Doldan P , Shahraz M , Schraivogel D , Gschwind AR , Sharma AK , Steinmetz LM *et al* (2021) Gene Expression Omnibus GSE156760 (https://www.ncbi.nlm.nih.gov/geo/query/acc.cgi?acc=GSE156760). [DATASET] 10.15252/msb.202110232 PMC8077299 33904651

[msb202110387-bib-0145] Türei D , Korcsmáros T , Saez‐Rodriguez J (2016) OmniPath: guidelines and gateway for literature‐curated signaling pathway resources. Nat Methods 13: 966–967 27898060 10.1038/nmeth.4077

[msb202110387-bib-0146] Türei D , Valdeolivas A , Gul L , Palacio‐Escat N , Klein M , Ivanova O , Ölbei M , Gábor A , Theis F , Módos D *et al* (2021) Integrated intra‐ and intercellular signaling knowledge for multicellular omics analysis. Mol Syst Biol 17 10.15252/msb.20209923 PMC7983032 33749993

[msb202110387-bib-0147] Urwyler P , Moser S , Charitos P , Heijnen IAFM , Rudin M , Sommer G , Giannetti BM , Bassetti S , Sendi P , Trendelenburg M *et al* (2020) Treatment of COVID‐19 with conestat alfa, a regulator of the complement, contact activation and kallikrein‐kinin system. Front Immunol 11: 2072 32922409 10.3389/fimmu.2020.02072 PMC7456998

[msb202110387-bib-0148] V’kovski P , Gerber M , Kelly J , Pfaender S , Ebert N , Braga Lagache S , Simillion C , Portmann J , Stalder H , Gaschen V *et al* (2019) Determination of host proteins composing the microenvironment of coronavirus replicase complexes by proximity‐labeling. eLife 8: e42037 30632963 10.7554/eLife.42037 PMC6372286

[msb202110387-bib-0149] Valencia I , Peiró C , Lorenzo Ó , Sánchez‐Ferrer CF , Eckel J , Romacho T (2020) DPP4 and ACE2 in diabetes and COVID‐19: therapeutic targets for cardiovascular complications? Front Pharmacol 11: 1161 32848769 10.3389/fphar.2020.01161 PMC7426477

[msb202110387-bib-0150] van den Berg DF , Te Velde AA (2020) Severe COVID‐19: NLRP3 inflammasome dysregulated. Front Immunol 11: 1580 32670297 10.3389/fimmu.2020.01580 PMC7332883

[msb202110387-bib-0151] Wang Y , An G , Becker A , Cockrell C , Collier N , Craig M , Davis CL , Faeder J , Versypt ANF , Gianlupi JF *et al* (2020) Iterative community‐driven development of a SARS‐CoV‐2 tissue simulator. BioRxiv 10.1101/2020.04.02.019075 [PREPRINT]

[msb202110387-bib-0152] Wei C‐H , Kao H‐Y , Lu Z (2015) GNormPlus: an integrative approach for tagging genes, gene families, and protein domains. BioMed Res Int 2015: 1–7 10.1155/2015/918710 PMC4561873 26380306

[msb202110387-bib-0153] Wilkinson MD , Dumontier M , IjJ A , Appleton G , Axton M , Baak A , Blomberg N , Boiten J‐W , da Silva Santos LB , Bourne PE *et al* (2016) The FAIR Guiding Principles for scientific data management and stewardship. Sci Data 3: 160018 26978244 10.1038/sdata.2016.18 PMC4792175

[msb202110387-bib-0154] Wimalaratne SM , Juty N , Kunze J , Janée G , McMurry JA , Beard N , Jimenez R , Grethe JS , Hermjakob H , Martone ME *et al* (2018) Uniform resolution of compact identifiers for biomedical data. Sci Data 5: 180029 29737976 10.1038/sdata.2018.29 PMC5944906

[msb202110387-bib-0155] Wolstencroft K , Krebs O , Snoep JL , Stanford NJ , Bacall F , Golebiewski M , Kuzyakiv R , Nguyen Q , Owen S , Soiland‐Reyes S *et al* (2017) FAIRDOMHub: a repository and collaboration environment for sharing systems biology research. Nucleic Acids Res 45: D404–D407 27899646 10.1093/nar/gkw1032 PMC5210530

[msb202110387-bib-0156] Wong HH , Fung TS , Fang S , Huang M , Le MT , Liu DX (2018) Accessory proteins 8b and 8ab of severe acute respiratory syndrome coronavirus suppress the interferon signaling pathway by mediating ubiquitin‐dependent rapid degradation of interferon regulatory factor 3. Virology 515: 165–175 29294448 10.1016/j.virol.2017.12.028 PMC7112132

[msb202110387-bib-0157] Xia S , Zhu Y , Liu M , Lan Q , Xu W , Wu Y , Ying T , Liu S , Shi Z , Jiang S *et al* (2020) Fusion mechanism of 2019‐nCoV and fusion inhibitors targeting HR1 domain in spike protein. Cell Mol Immunol 17: 765–767 32047258 10.1038/s41423-020-0374-2 PMC7075278

[msb202110387-bib-0158] Xiong R , Zhang L , Li S , Sun Y , Ding M , Wang Y , Zhao Y , Wu Y , Shang W , Jiang X *et al* (2020) Novel and potent inhibitors targeting DHODH are broad‐spectrum antivirals against RNA viruses including newly‐emerged coronavirus SARS‐CoV‐2. Protein Cell 11: 723–739 32754890 10.1007/s13238-020-00768-w PMC7402641

[msb202110387-bib-0159] Yang N , Shen H‐M (2020) Targeting the endocytic pathway and autophagy process as a novel therapeutic strategy in COVID‐19. Int J Biol Sci 16: 1724–1731 32226290 10.7150/ijbs.45498 PMC7098027

[msb202110387-bib-0160] Zhang H , Tu J , Cao C , Yang T , Gao L (2020) Proteasome activator PA28γ‐dependent degradation of coronavirus disease (COVID‐19) nucleocapsid protein. Biochem Biophys Res Commun 529: 251–256 32703419 10.1016/j.bbrc.2020.06.058 PMC7296323

[msb202110387-bib-0161] Zhang X , Ding M , Zhu P , Huang H , Zhuang Q , Shen J , Cai Y , Zhao M , He Q (2019) New insights into the Nrf‐2/HO‐1 signaling axis and its application in pediatric respiratory diseases. Oxid Med Cell Longev 2019: 3214196 31827672 10.1155/2019/3214196 PMC6885770

[msb202110387-bib-0162] Zheng Y , Zhuang M‐W , Han L , Zhang J , Nan M‐L , Zhan P , Kang D , Liu X , Gao C , Wang P‐H (2020) Severe acute respiratory syndrome coronavirus 2 (SARS‐CoV‐2) membrane (M) protein inhibits type I and III interferon production by targeting RIG‐I/MDA‐5 signaling. Signal Transduct Target Ther 5: 299 33372174 10.1038/s41392-020-00438-7 PMC7768267

[msb202110387-bib-0163] Ziegler CGK , Allon SJ , Nyquist SK , Mbano IM , Miao VN , Tzouanas CN , Cao Y , Yousif AS , Bals J , Hauser BM *et al* (2020) SARS‐CoV‐2 receptor ACE2 is an interferon‐stimulated gene in human airway epithelial cells and is detected in specific cell subsets across tissues. Cell 181: 1016–1035 32413319 10.1016/j.cell.2020.04.035 PMC7252096

[msb202110387-bib-0164] Zipeto D , da Palmeira JF , Argañaraz GA , Argañaraz ER (2020) ACE2/ADAM17/TMPRSS2 interplay may be the main risk factor for COVID‐19. Front Immunol 11: 576745 33117379 10.3389/fimmu.2020.576745 PMC7575774

