# Supplementary material for: COVID19 Disease Map, a computational knowledge repository of virus–host interaction mechanisms
Source: Mol Syst Biol. 2021 Oct 19;17(10):e10387. doi: 10.15252/msb.202110387 (PMC8524328; doi:10.15252/msb.202110387)
Supplement: Supplementary file 2 — Expanded View Figures PDF [file MSB-17-e10387-s002.pdf]

Expanded View Figures

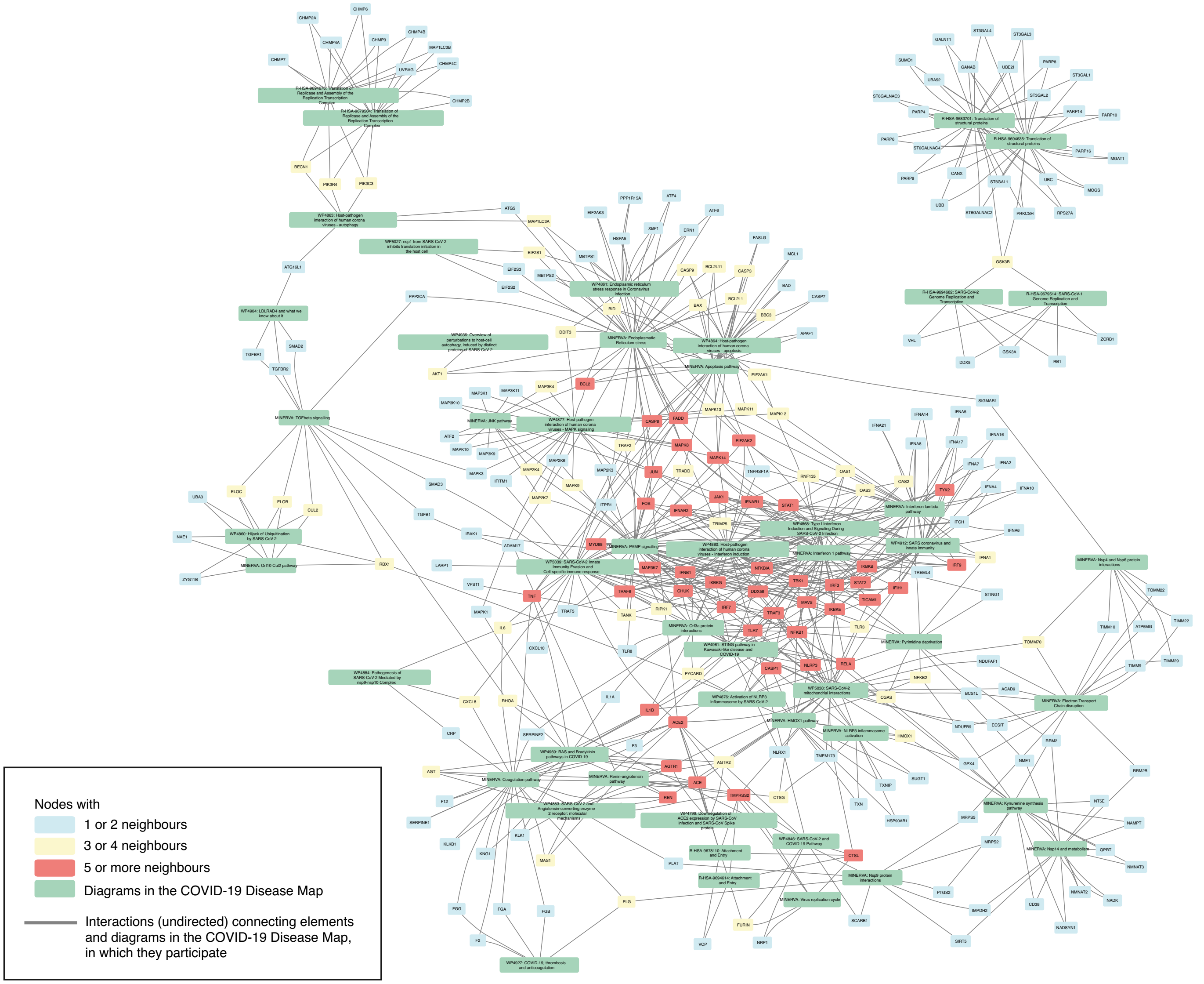

Figure EV1. Exploration of the existing crosstalk between the diagrams of the COVID-19 Disease Map.

The network structure of the diagrams and their interactions based on existing crosstalk (shared elements). Colour code: green—pathways; blue—proteins with one or two neighbours; yellow—proteins with three or four neighbours; and red—proteins with five or more neighbours. See Materials and Methods for details.

© 2021 The Authors

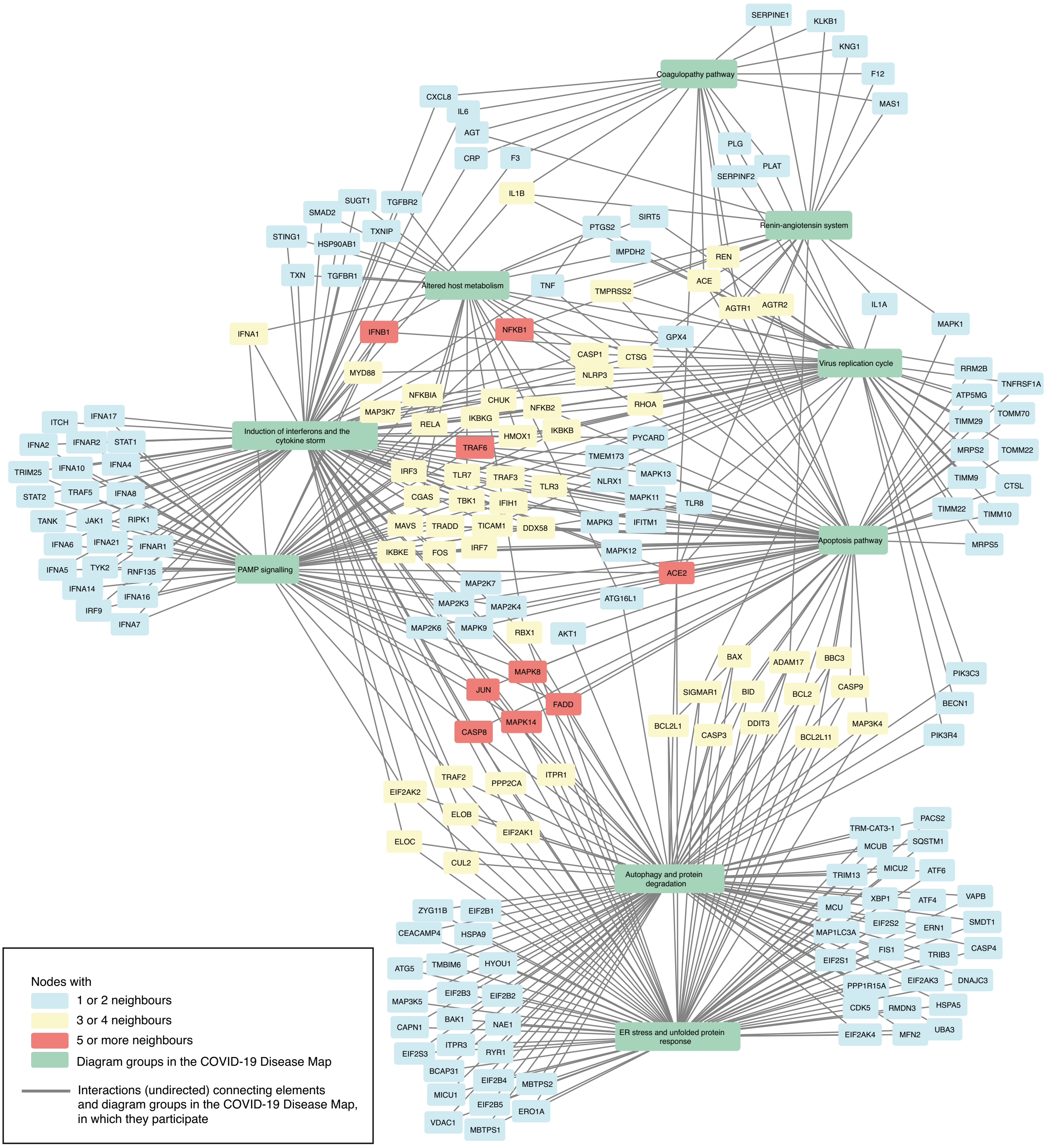

Figure EV2. Exploration of the existing crosstalk between the groups of diagrams of the COVID-19 Disease Map.

The network structure of the diagrams and their interactions based on existing crosstalk (shared elements). Colour code: green—pathway groups; blue—proteins with one or two neighbours, yellow—proteins with three or four neighbours; and red—proteins with five or more neighbours. See Materials and Methods for details.

© 2021 The Authors

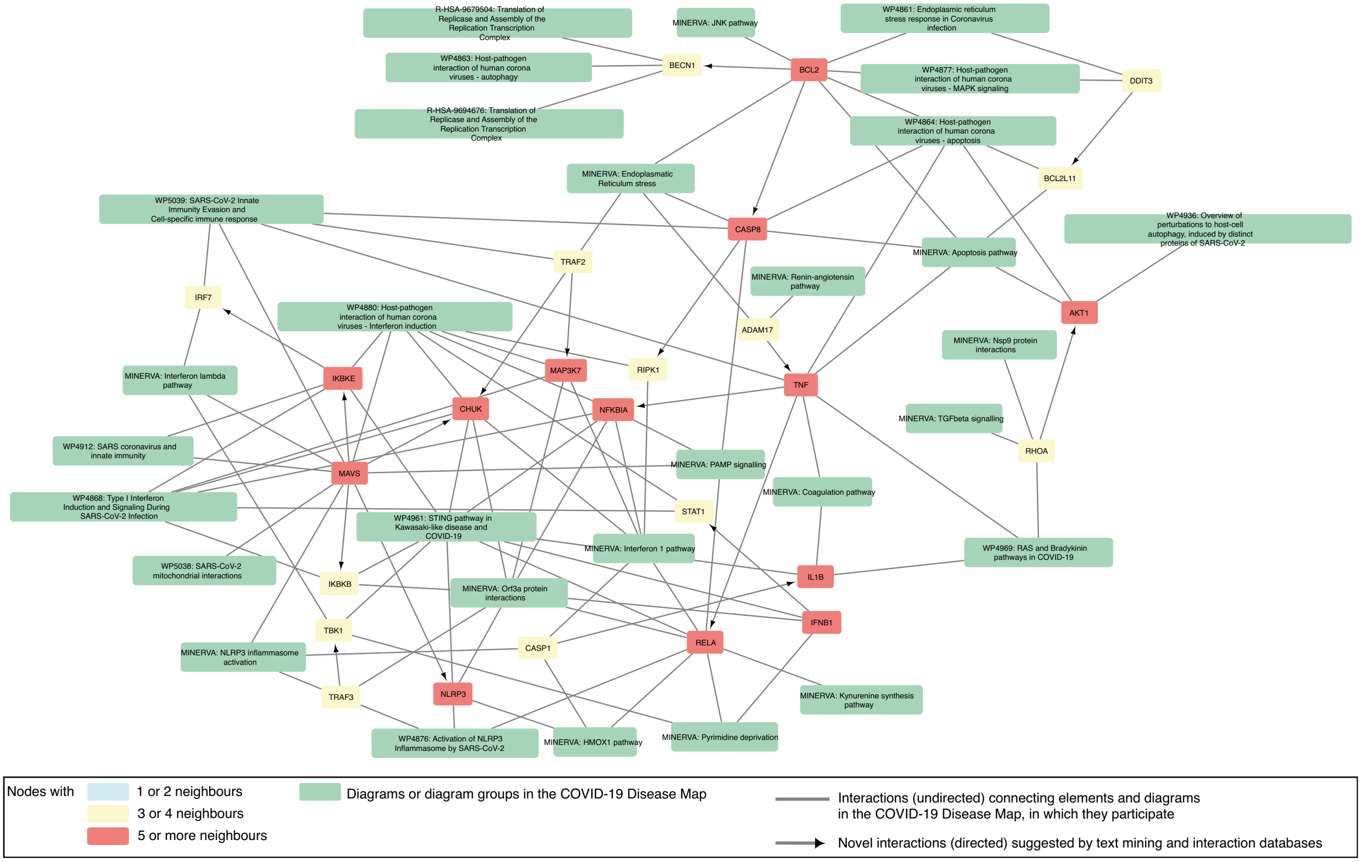

Figure EV3. Exploration of new crosstalk between the diagrams of the COVID-19 Disease Map.

The network structure of the diagrams and their interactions based on new crosstalk. Colour code: green—pathways; blue—proteins with one or two neighbours; yellow—proteins with three or four neighbours; and red—proteins with five or more neighbours. New molecular interactions are shown as directed edges. See Materials and Methods for details.

© 2021 The Authors
