# Supplementary material for: COVID19 Disease Map, a computational knowledge repository of virus–host interaction mechanisms
Source: Mol Syst Biol. 2021 Oct 19;17(10):e10387. doi: 10.15252/msb.202110387 (PMC8524328; doi:10.15252/msb.202110387)
Supplement: Supplementary file 3 — Table EV1 [file MSB-17-e10387-s004.docx]

**Table EV1. COVID-19 Disease Map diagrams.** The table summarises the main diagrams of the C19DMap project, their scope and how to access them. HCoV - Human Coronavirus. Available online at <https://covid.pages.uni.lu/map_contents>.

| **Diagram groups and diagrams** | **Diagrams: Resource** | **Diagrams:  Access** | **Virus proteins** |
| --- | --- | --- | --- |
| **Virus replication cycle:**  Attachment and entry | WikiPathways | [WP4846](https://www.wikipathways.org/index.php/Pathway:WP4846) | All |
|  | Reactome | [R-HSA-9678110](https://reactome.org/PathwayBrowser/#/R-HSA-9678108&SEL=R-HSA-9678110), [R-HSA-9694614](https://reactome.org/PathwayBrowser/#/R-HSA-9694516&SEL=R-HSA-9694614) |  |
|  | MINERVA (gitlab) | [Virus replication cycle](https://covid19map.elixir-luxembourg.org/minerva/?search=virus%20replication%20cycle) ([gitlab](https://git-r3lab.uni.lu/covid/models/-/tree/master/Curation/Virus%20replication%20cycle)) |  |
| **Virus replication cycle:**  Transcription, translation and replication | WikiPathways | [WP4846](https://www.wikipathways.org/index.php/Pathway:WP4846) | All |
|  | Reactome | [R-HSA-9679504](https://reactome.org/PathwayBrowser/#/R-HSA-9678108&SEL=R-HSA-9679504), [R-HSA-9694676](https://reactome.org/PathwayBrowser/#/R-HSA-9694516&SEL=R-HSA-9694676)  [R-HSA-9679514](https://reactome.org/PathwayBrowser/#/R-HSA-9678108&SEL=R-HSA-9679514), [R-HSA-9694682](https://reactome.org/PathwayBrowser/#/R-HSA-9694516&SEL=R-HSA-9694682)  [R-HSA-9683701](https://reactome.org/PathwayBrowser/#/R-HSA-9678108&SEL=R-HSA-9683701), [R-HSA-9694635](https://reactome.org/PathwayBrowser/#/R-HSA-9694516&SEL=R-HSA-9694635) |  |
|  | MINERVA (gitlab) | [Virus replication cycle](https://covid19map.elixir-luxembourg.org/minerva/?search=virus%20replication%20cycle) ([gitlab](https://git-r3lab.uni.lu/covid/models/-/tree/master/Curation/Virus%20replication%20cycle))  [RTC and transcription](https://covid19map.elixir-luxembourg.org/minerva/?search=sars-cov-2%20rtc%20and%20transcription) ([gitlab](https://git-r3lab.uni.lu/covid/models/-/tree/master/Curation/RTC-and-transcription))  [Nsp9 interactions](https://covid19map.elixir-luxembourg.org/minerva/?search=nsp9%20protein%20interactions) ([gitlab](https://git-r3lab.uni.lu/covid/models/-/tree/master/Curation/SARS-CoV-2%20proteins/Nsp9))  [E protein interactions](https://covid19map.elixir-luxembourg.org/minerva/?search=e%20protein%20interactions) ([gitlab](https://git-r3lab.uni.lu/covid/models/-/tree/master/Curation/SARS-CoV-2%20proteins/E)) |  |
| **Virus replication cycle:**  Assembly and release | Reactome | [R-HSA-9679509](https://reactome.org/PathwayBrowser/#/R-HSA-9678108&SEL=R-HSA-9679509), [R-HSA-9694322](https://reactome.org/PathwayBrowser/#/R-HSA-9694516&SEL=R-HSA-9694322) | All |
|  | MINERVA (gitlab) | [Virus replication cycle](https://covid19map.elixir-luxembourg.org/minerva/?search=virus%20replication%20cycle) ([gitlab](https://git-r3lab.uni.lu/covid/models/-/tree/master/Curation/Virus%20replication%20cycle))  [Nsp4 and Nsp6 interactions](https://covid19map.elixir-luxembourg.org/minerva/?search=nsp4%20and%20nsp6%20protein%20interactions) ([gitlab](https://git-r3lab.uni.lu/covid/models/-/tree/master/Curation/SARS-CoV-2%20proteins/Nsp4_Nsp6)) |  |
| **Viral subversion of host defence:**  ER stress and unfolded protein response | WikiPathways | [WP4861](https://www.wikipathways.org/index.php/Pathway:WP4861) | S (HCoV OC43)  4a (MERS)  Orf8ab, E, nsp15 (SARS-CoV-1) |
|  | MINERVA (gitlab) | [ER stress](https://covid19map.elixir-luxembourg.org/minerva/?search=endoplasmatic%20reticulum%20stress) ([gitlab](https://git-r3lab.uni.lu/covid/models/-/tree/master/Curation/ER%20Stress)) |  |
| **Viral subversion of host defence:**  Autophagy and protein degradation | WikiPathways | [WP4860](https://www.wikipathways.org/index.php/Pathway:WP4860), [WP4936](https://www.wikipathways.org/index.php/Pathway:WP4936), [WP4863](https://www.wikipathways.org/index.php/Pathway:WP4863) | nsp6 (SARS-CoV-1)  nsp567 (PRRSV)  Nsp6, Orf3, Orf10 (SARS-CoV-2) |
|  | MINERVA (gitlab) | [Orf10 Cul2 pathway](https://covid19map.elixir-luxembourg.org/minerva/?search=Orf10%20Cul2%20pathway) ([gitlab](https://git-r3lab.uni.lu/covid/models/-/tree/master/Curation/Ubiquination%20and%20Protein%20Degradation)) |  |
| **Viral subversion of host defence:**  Apoptosis | WikiPathways | [WP4864](https://www.wikipathways.org/index.php/Pathway:WP4864) | Orf3a, Orf3b, Orf6, Orf8a, Orf7a, Orf9b, E, M, N, S (SARS-CoV-1)  Nsp4. Nsp5, Nsp6. Nsp7, Nsp8, Orf9c (SARS-CoV-2) |
|  | MINERVA (gitlab) | [Apoptosis pathway](https://covid19map.elixir-luxembourg.org/minerva/?search=apoptosis%20pathway) ([gitlab](https://git-r3lab.uni.lu/covid/models/-/tree/master/Curation/Apoptosis))  [JNK pathway](https://covid19map.elixir-luxembourg.org/minerva/?search=jnk%20pathway) ([gitlab](https://git-r3lab.uni.lu/covid/models/-/tree/master/Curation/JNK%20pathway))  [ETC disruption](https://covid19map.elixir-luxembourg.org/minerva/?search=electron%20transport%20chain%20disruption) ([gitlab](https://git-r3lab.uni.lu/covid/models/-/tree/master/Curation/ETC)) |  |
| **Integrative stress response:**  Renin-angiotensin system | WikiPathways | [WP4883](https://www.wikipathways.org/index.php/Pathway:WP4883), [WP4799](https://www.wikipathways.org/index.php/Pathway:WP4799), [WP4965](https://www.wikipathways.org/index.php/Pathway:WP4965) | S (SARS-CoV-2) |
|  | MINERVA (gitlab) | [Renin-angiotensin pathway](https://covid19map.elixir-luxembourg.org/minerva/?search=renin-angiotensin%20pathway) ([gitlab](https://git-r3lab.uni.lu/covid/models/-/tree/master/Curation/Renin-angiotensin%20pathway)) |  |
| **Integrative stress response:**  Coagulopathy | WikiPathways | [WP4927](https://www.wikipathways.org/index.php/Pathway:WP4927) | S (SARS-CoV-2) |
|  | MINERVA (gitlab) | [Coagulation pathway](https://covid19map.elixir-luxembourg.org/minerva/?search=Coagulation%20pathway) ([gitlab](https://git-r3lab.uni.lu/covid/models/-/tree/master/Curation/Coagulation%20pathway)) |  |
| **Innate Immune Response:**  PAMP signalling | WikiPathways | [WP4912](https://www.wikipathways.org/index.php/Pathway:WP4912) | nsp3, nsp15, Orf3b, Orf8, Orf9, M, N, S (SARS-CoV-1) |
|  | MINERVA | [PAMP signalling](https://covid19map.elixir-luxembourg.org/minerva/?search=PAMP%20signalling) ([gitlab](https://git-r3lab.uni.lu/covid/models/-/tree/master/Curation/PAMP%20signalling)) |  |
| **Innate Immune Response:**  Induction of interferons and the cytokine storm | WikiPathways | [WP4868](https://www.wikipathways.org/index.php/Pathway:WP4868), [WP4880](https://www.wikipathways.org/index.php/Pathway:WP4880), [WP4876](https://www.wikipathways.org/index.php/Pathway:WP4876) | 4a, 4b, PLPro, S (MERS)  nsp3, nsp13, Orf3a, Orf6, Orf7a, Orfab, Orfb, M, N, S (SARS-CoV-1) |
|  | MINERVA | [Interferon I pathway](https://covid19map.elixir-luxembourg.org/minerva/?search=interferon%201%20pathway) |  |
| **Innate Immune Response:**  Altered host metabolism | WikiPathways | [WP4853](https://www.wikipathways.org/index.php/Pathway:WP4853) | Nsp14, Orf3a, E, M, N, S (SARS-CoV-2) |
|  | MINERVA (gitlab) | [Heme Oxygenase pathway](https://covid19map.elixir-luxembourg.org/minerva/?search=hmox1%20pathway) ([gitlab](https://git-r3lab.uni.lu/covid/models/-/tree/master/Curation/HMOX1%20pathway))  [Kynurenine synthesis pathway](https://covid19map.elixir-luxembourg.org/minerva/?search=kynurenine%20synthesis%20pathway) ([gitlab](https://git-r3lab.uni.lu/covid/models/-/tree/master/Curation/Kynurenine%20Pathway))  [Amino sugar and nucleotide sugar metabolism](https://covid19map.elixir-luxembourg.org/minerva/?search=nsp14%20and%20metabolism) ([gitlab](https://git-r3lab.uni.lu/covid/models/-/tree/master/Curation/SARS-CoV-2%20proteins/Nsp14))  [Pyrimidine deprivation pathway](https://covid19map.elixir-luxembourg.org/minerva/?search=pyrimidine%20deprivation) ([gitlab](https://git-r3lab.uni.lu/covid/models/-/tree/master/Curation/Pyrimidine%20deprivation)) |  |
