# Supplementary material for: COVID19 Disease Map, a computational knowledge repository of virus–host interaction mechanisms
Source: Mol Syst Biol. 2021 Oct 19;17(10):e10387. doi: 10.15252/msb.202110387 (PMC8524328; doi:10.15252/msb.202110387)
Supplement: Supplementary file 4 — Table EV2 [file MSB-17-e10387-s003.docx]

**Table EV2.** HiPathia results for the Apoptosis diagram of the COVID-19 Disease Map.

| **Pathway:circuit name** | **UP/DOWN**  **regulation** | **statistic** | **p.value** | **FDRp.value** | **Fold_Change** | **logFC** |
| --- | --- | --- | --- | --- | --- | --- |
| Apoptosis: CASP7 | DOWN | -2,081 | 0,095 | 0,037 | 0,398 | -1,330 |
| Apoptosis: CASP3 | DOWN | -2,352 | 0,054 | 0,019 | 0,370 | -1,436 |
